# The potential of mitochondrial permeability transition-driven necrosis-related genes in prognostic evaluation of colorectal cancer patients

**DOI:** 10.3389/fonc.2026.1679360

**Published:** 2026-03-09

**Authors:** ChenXin Huang, DiYa Xie, BinSheng Lin, Kun Zhang

**Affiliations:** Department of General Surgery, Fuzhou First General Hospital Affiliated with Fujian Medical University, Fuzhou, Fujian, China

**Keywords:** colorectal cancer, mitochondrial permeability transition driven necrosis, prognostic genes, risk model, single-cell analysis

## Abstract

**Background:**

Mitochondrial permeability transition-driven necrosis (MPTDN) has been implicated in a variety of diseases, but its relationship with colorectal cancer (CRC) prognosis is unclear.

**Methods:**

In this study, TCGA-COAD and TCGA-READ datasets were analyzed to identify differentially expressed genes (DEGs) in CRCs. Differentially expressed MPTDNGRs (DE-MPTDNRGs) were identified by comparing DEGs to MPTDN-related genes (MPTDNRGs). Univariate Cox, Minimum Absolute Contraction and Selection Operator (LASSO) regression and risk scoring analysis divided TCGA-CRC patients into high- and low-risk cohorts.

**Results:**

The prognostic genes *LMNB2*, *CASP7*, *PRKCB*, *GZMB* and *ENDOG* were identified, and the survival rate of high-risk patients was poor. Independent prognostic factors, including risk score, age, and N stage, are effective predictors of survival. Immunoassays revealed that high-risk patients had 9 elevated immune checkpoints, while low-risk patients were more susceptible to pazopanib and temsirolimus. In addition, single-cell analysis showed that *PRKCB* and *GZMB* were highly expressed in stem cells, while *LMNB2* was more abundantly expressed in mast cells. Real-time PCR (RT-qPCR) confirmed low levels of *CASP7*, *PRKCB*, and *ENDOG* mRNA in CRC tissues, with no significant difference between *LMNB2* and *GZMB*.

**Conclusion:**

These findings highlight 5 MPTDN-associated prognostic genes in CRC, providing insights for individualized treatment and prognosis.

## 1 Introduction

Colorectal cancer (CRC) is still a dominant reason for tumor incidence and mortality worldwide (1). The global burden of colorectal cancer disease on society is expected to increase significantly by 2040, with new cases rising to 3.2 million and deaths rising to 1.6 million (2). Current diagnostic methods for CRC, including colonoscopy and fecal immunochemical tests, are effective but have limitations such as invasiveness, cost, and patient compliance issues. New agents targeting KRAS G12C mutation mark a paradigm shift in terms of managing the influenced patients and may drive progress in developing treatments for the more prevalent KRAS mutations (3). Novel innovative combinations with poly (ADP-ribose) polymerase (PARP) suppressors can advance the existing curative landscape (4). Despite progress in screening and therapeutic strategies guided by biomarker testing for BRAF mutations, deficient Mismatch Repair (dMMR), microsatellite instability (MSI), and HER2 amplification (5), the prognosis for advanced CRC is still compromised by suboptimal responses. This highlights the intricate heterogeneity of CRC and underscores the imperative for the development of innovative diagnostic and therapeutic modalities (6).

Mitochondrial Permeability Transition-driven necrosis (MPTDN) represents the regulated form of cell death (RCD) pathway that is induced by oxidative stress and disruptions in the cellular environment. It causes the rapid loss of mitochondrial membrane potential and cell membrane rupture (7). MPTDN is implicated in diseases like ischemia-reperfusion injury, neurodegenerative diseases, acute kidney injury, and melanoma (8). It involves mitochondrial permeability transition pore (mPTP) opening, causing mitochondrial dysfunction and cell death. For instance, Icaritin, a natural compound, inhibits CRC cell proliferation by activating the JNK signaling pathway and inducing mPTP opening, thereby triggering necrosis (9). Other therapeutic agents, such as Curcumin, leverage this pathway (10), utilizing mPTP blockers like CsA and SfA to attenuate necrosis. Dynamin-related protein 1 (DRP1), an important effector protein in mitochondrial fission, exerts a dual role in the development of CRC (11). On one hand, DRP1-mediated mitochondrial fission is instrumental in metabolic reprogramming within CRC cells, which is essential for the regulation of fatty acids and glucose metabolism. This reprogramming not only enhances cell proliferation, invasion, and migration but also contributes to chemoresistance. On the other hand, the activation of DRP1 also triggers carcinogenic signaling pathways, like the Wnt/β-catenin pathway that fosters cell progress and migration in CRC. These findings suggest that MPTDN-related genes (MPTDNRGs) have the potential for acting as biomarkers and therapeutic targets for CRC, offering possible clinical value. Exploring the roles of other MPTDNRGs in CRC could provide novel perceptions of early diagnosis, therapy, and individualized medicine approaches in such disease.

MPTDN is a well-established mechanism implicated in ischemia-reperfusion injury and neurodegenerative disorders. However, its systematic role in the progression of CRC and its potential as a prognostic marker remain largely unexplored. Currently, there is no comprehensive prognostic classifier based on MPTDN-related genes for CRC, and the influence of these genes on the immune microenvironment and therapeutic responsiveness has not been thoroughly investigated. To address these gaps, we conducted an integrative analysis using data from The Cancer Genome Atlas (TCGA), Gene Expression Omnibus (GEO), and single-cell transcriptomic datasets, complemented by in-house quantitative PCR (qPCR) data. This approach enabled us to develop and externally validate the first MPTDN-based risk signature, explore its immunophenotypic implications, and assess its correlation with drug response. By translating MPTDN biology into a practical stratification tool, we offer a mechanistically informed framework for enhancing prognosis and designing combination therapies in CRC.

## 2 Materials and methods

### 2.1 Data extraction

RNA-seq data were obtained from the Cancer Genome Atlas (TCGA) database (https://portal.gdc.cancer.gov/) for CRC specimens, specifically, the TCGA-COAD and TCGA-READ datasets were combined to create a unified dataset known as TCGA-CRC. A total of 701 samples were gathered from 627 patients, encompassing 650 samples of tumor tissue and 51 samples of normal tissue. Out of these samples, survival information was accessible for 322 patients. The integration method of TCGA-COAD and TCGA-READ datasets was as follows:GDCquery() was executed separately for TCGA-COAD and TCGA-READ to construct query objects, followed by data downloading via the API using GDCdownload(method=“api”). The downloaded files were organized into SummarizedExperiment objects with GDCprepare().The count matrix (RDrr) was extracted from the SummarizedExperiment using assay() (yielding raw counts in a gene × sample format). Gene annotation information (e.g., gene_type, gene_name) was retrieved via rowRanges() and merged with the count matrix, ultimately generating annotated count matrices for COAD and READ respectively. After aligning at the gene level, samples from the two datasets were combined into a unified expression matrix for downstream analyses.For clinical data, GDCquery(data.category=“Clinical”, data.type=“Clinical Supplement”, data.format=“BCR Biotab”) was applied to TCGA-COAD and TCGA-READ individually to obtain clinical supplement information, which was then downloaded and parsed using GDCdownload() and GDCprepare(…, summarizedExperiment=TRUE) to generate clinical tables directly usable for survival analysis. A total of 268 samples with available clinical information were included.

The GSE39582, GSE282542 and GSE132465 datasets were supplied by the Gene Expression Omnibus (GEO) database (https://www.ncbi.nlm.nih.gov/gds). The GSE39582 dataset comprised 585 tumor tissue specimens, of which 579 samples had survival information. The GSE282542 dataset contained 8 colorectal cancer samples. The GSE132465 dataset comprises 23 tissue samples from patients with primary colorectal cancer (CRC) and 10 normal mucosal samples. A compilation of genes associated with mitochondrial permeability transition (MPT) originated in the Molecular Signatures Database (MSigDB, https://www.gsea-msigdb.org/gsea/msigdb/index.jsp), with the gene sets M17902, M3873, and M16257, which provided a total of 39 MPTDNRGs (12).

### 2.2 Difference analysis

To detect differentially expressed genes (DEGs) in the TCGA-CRC dataset (tumor vs. normal), the DESeq2 package was employed (p-value< 0.05 & |log_2_FC (fold change)| > 1) (v 1.34.0) (13). The P-value was through the Benjamini-Hochberg (BH). The results were visualized via heatmap and volcano plot.

### 2.3 Function analysis

The DEGs and MPTDNRGs were intersected to obtain DE-MPTDNRGs. To explore the function of DE-MPTDNRGs, gene ontology (GO) and Kyoto Encyclopedia of Genes and Genes (KEGG) pathway enrichment analyses were conducted through the “clusterprofiler” package (p-value< 0.05) (v 4.0.5) (14). The P-value was corrected through the BH. The results were visualized via heatmaps and volcano plots.

### 2.4 Construction and validation of the risk model

Using 322 tumor samples showing survival data form the TCGA-CRC dataset as the training set, univariate Cox regression (HR≠1, p-value< 0.05) was performed for DE-MPTDNRGs to detect prognostic related genes employing coxph function in survival package (v 3.2-13) (15) Subsequently, genes with P values greater than 0.05 were screened by PH hypothesis test for subsequent analysis. The proportional hazards (PH) assumption was tested using the Grambsch–Therneau test based on scaled Schoenfeld residuals via survival::cox.zph(), with the reporting of test P-values for each covariate as well as the global test (GLOBAL). Additionally, survminer::ggcoxzph() was utilized to generate smoothed curves of Schoenfeld residuals over time with confidence bands for visual diagnosis. Genes with P values greater than 0.05 were screened by PH hypothesis test for subsequent analysis. Subsequently, the glmnet package (v4.1-2) was employed to perform LASSO regression analysis on the identified prognostic genes (x, y, family=“cox”, maxit=5000, K = 10) (16). For the Cox model, cv.glmnet defaults to using the deviance of partial likelihood as the cross-validation evaluation metric (type.measure = “deviance”).The formula was as follows:


$$RS=\sum_{i=1}^{n}ei\times wi$$

Where N is the number of genes, e is the expression level, and w is the LASSO coefficient of the prognostic genes (17). The risk score calculation formula provides sparse solutions through L1 regularization, making it suitable for high-dimensional biological data. It ensures good predictive performance and generalizability through cross-validation, while simplifying the model for clinical translation applications. Patients were stratified into high and low-risk cohorts in light of the median risk score. Initially, survival differences between these groups were contrasted through Kaplan-Meier (K-M) curves, which were generated with the survminer package (v 0.4.9) (18). Simultaneously, the performance of predicting the survival status of patients was evaluated using receiver operating characteristic (ROC) curves produced with the survivalROC package (v 1.0.3) (19) for different time points. Finally, the constructed risk model was verified through the validation set GSE39582. Additionally, Based on the optimal cut-off values of biomarker expression, we divided the patient samples into high and low expression groups, and analyzed the survival differences between these two groups using the survminer package (v 0.4.9).

### 2.5 Screening of independent prognostic factors

Initially, the PH assumption test (p > 0.05) was performed for the risk score and clinical characteristics (age, Clinical Stage, T Stage, N Stage, M Stage) in the TCGA-CRC dataset. Subsequently, univariate Cox regression analysis (HR ≠ 1, P< 0.05) was conducted using the survival package (v 3.2-13) to screen for prognostic factors associated with clinical characteristics and the risk score. Finally, the factors passing the univariate Cox analysis underwent the PH assumption test (p > 0.05) and multivariate Cox regression analysis (HR ≠ 1, P< 0.05), leading to the identification of the final independent prognostic factors (IPF).

### 2.6 Construction of a nomogram

The rms package (v 6.2-0) (20) was employed to develop a nomogram incorporating IPFs for predicting the survival probability of patients at different years. Calibration curves and ROC were drawn for the performance evaluation of the nomogram via survivalROC package (v 1.0.3) (AUC > 0.6). Additionally, decision curve analysis (DCA) curves were plotted with the rmda package (v 1.6) to assess the clinical utility of the model. To confirm the unique value of this prognostic model, a direct comparison was conducted with previously published models (21–25)in the TCGA-CRC dataset, with consistency index (Cindex) > 0.6 and AUC > 0.6 as evaluation criteria. Corresponding ROC curves were also plotted (AUC > 0.6).

### 2.7 Gene set enrichment analysis

As to the training set, to explore potential differences in function between two risk cohorts, DEGs between two risk cohorts were screened with the DESeq2 package (p-value< 0.05 & |log2FC| > 1) (13). The P-value was corrected through the BH. Additionally, to explore their potential functions, correlation coefficients between prognostic genes and all genes in the training set were calculated and sequenced. Secondly, in c5.go.bp.v7.2.symbols. gmt and c2.ccp.kegg.v7.2.symbols.gmt as background gene set, clusterProfiler package was used to carry out GSEA enrichment analysis (p.adjust< 0.05) (v 4.0.5) (14).

### 2.8 Processing and quality control of single cell RNA sequencing data

The single-cell transcriptomic sequencing data (GSE282542) was constructed into Seurat objects through the R package “Seurat,”(v 5.1.0) (26) with cells having less than 200 genes. Moreover, genes covered by less than three cells excluded. The inclusion criteria were specified as follows: nFeature RNA (the number of genes quantified per cell) was required to fall within the range of 200 to 6,000, nCount RNA (the total gene counts per cell) was restricted to less than 8,000, and percentmt (the proportion of mitochondrial gene expression per cell) was restricted to less than 20%. Doublets were removed using the R package “scDblFinder” (v 1.16.0) (27). The data was then normalized using the NormalizeData function from the “Seurat” package (v 5.1.0). The parameters were initialized to “LogNormalize” and scale.factor was initialized to 10000. The FindVariableFeatures function was employed to identify highly variable genes based on the correlation between the mean and variance of gene expression, with the top 2,000 highly variable genes selected for further analysis by default (selection.method = “vst”). Results were observed through the LabelPoints function, with the top 10 most variable genes labeled. To eliminate technical variation among samples, the Harmony algorithm was employed for batch effect correction. Batch effects were evaluated using scatter plots after principal component analysis (PCA) dimensionality reduction, and the DimPlot function was utilized to visualize the distribution of different samples in the PCA space. Subsequently, the Harmony algorithm was run for data integration, with sample origin (orig.ident) as the grouping variable and the theta parameter set to 6 to balance the integration intensity.

To further confirm and analyze the cell populations of different cell groups, the R package “Seurat”(v 5.1.0) was applied to standardize the single-cell transcriptome sequencing data (GSE282542) through the ScaleData function. Subsequently, PCA was executed on the highly variable genes of each sample for dimensionality reduction. The Jackstraw function was then used to generate a Jackstraw plot, and the re-clustering was performed using a permutation test algorithm to select the appropriate principal components (100 simulations were repeated to calculate the p-values of the principal components, with a significance threshold set at p< 0.05). The ElbowPlot function was used to create an elbow plot to identify the usable dimensions. Based on the results showing a plateau in the PCA feature number and strong differentiation capability, combined with the analysis from the Jackstraw plot, suitable principal components were selected for further analysis. The FindNeighbors and FindClusters functions in Seurat (v 5.1.0) were used to identify different cell clusters, and uniform manifold approximation and projection (UMAP) was applied to visualization (resolution = 0.2). For cell type annotation within the clusters, the SingleR method (v 1.831) (26) and manual labeling were used to annotate each cell cluster. Finally, UMAP plots of the annotated cell clusters were generated, and a DotPlot was formulated to show the distribution of prognostic genes across discrepant groups and cell types. Meanwhile, the expression of prognostic genes across different cell types was validated in the GSE132465 dataset. Since the dataset downloaded from the GEO database had already undergone QC, no additional QC was performed (28).

### 2.9 Immune analysis

Via estimating relative subsets of RNA transcripts (CIBERSORT), cell type recognition was utilized to assess immune cell infiltration by evaluating the relative percentage of 22 immune cell types across all samples in the training set (p< 0.05). The P-value was corrected through the BH. Samples with immune infiltration values exceeding 0.05 and immune cell types with no infiltration were excluded. Additionally, the xCell algorithm within the IOBR package (v2.0.0) (29) was utilized to calculate the infiltration abundances of 44 immune cell types in CRC samples from the high- and low-risk cohorts (p< 0.05). In addition, similar methods were applied to compare immune checkpoint gene expression between the two risk cohorts. Finally, through the tumor immune dysfunction and exclusion (TIDE) website (http://tide.dfci.harvard.edu/) to figure out the TIDE scores for two risk cohorts of patients and analyze the difference (p< 0.05). To assess the microsatellite instability (MSI) status in the training set, the cBioPortalData package (v2.14.0) (30) was used to download MSI scores. A score of “MSI_SENSOR_SCORE” greater than 0.3 was used to define MSI, while scores equal to or below 0.3 were defined as microsatellite stable (MSS). The differences in risk scores between the MSI and MSS cohorts were comparedTo predict the response to immunotherapy, the cancer immunome atlas the Cancer Imaging Archive (TCIA, https://tcia.at/) was utilized to obtain the immune prognostic score (IPS) of TCGA-CRC patients, and subsequently, the disparities in IPS between two risk cohorts were compared. Spearman correlation was employed to calculate the correlation (|cor| > 0.3, p< 0.05).

### 2.10 Drug sensitivity analysis

The pRRophetic package (v 0.5) (31) was utilized to compute the Half Maximal Inhibitory Concentration (IC50) for each tumor sample in the training set. IC50 values were employed to assess patients’ sensitivity to drug therapy (p< 0.05).

### 2.11 The protein level analysis of key prognostic genes

In order to further construe the potential relevance between prognostic genes and CRC at the protein level, we adopted the Human Protein Atlas (HPA) online database (http://www.proteinatlas.org/) to analyze the protein expression levels of prognostic genes in CRC tissue specimens.

### 2.12 Ethical approval and consent to participate

Five CRC patients and five healthy individuals’ tissue samples were gathered by the Fuzhou First General Hospital following the principles of the Helsinki Declaration and approved by the Fuzhou First Hospital Ethics Committee (Ethics Review Number: 202312065).

### 2.13 RT-qPCR

Total RNA was obtained from the samples using TRIzol reagent as per the protocol. After that, RNA concentration was determined through NanoDrop, and mRNA was converted into cDNA by the SweScript First Strand cDNA Synthesis Kit. Following this, SYBR Green reagent, cDNA, and forward and reverse primers were mixed for the RT-qPCR reaction (**Table 1**). RT-qPCR amplification was executed more than 40 cycles, with each assay conducted in triplicate. The relative levels of gene mRNA were assessed utilizing the 2^–△△Ct^ method in compliance with GAPDH employed as the internal reference. This has been approved by the relevant ethics committee of Fuzhou First General Hospital. The approval number and date of approval are as follows: [202312010] and [Dec. 27th, 2023].

**Table 1:** RT-qPCR primer sequence list.

Primer	Sequence
*LMNB2* F	5’-CCTGACTCCTATCACGACGC-3’
*LMNB2* R	5’-GGGAGGGTGTAAACGGTGAG-3’
*CASP7* F	5’-CGCAAAGCAACGTCTAGGAG-3’
*CASP7* R	5’-GAATCCTCAACCCCCTGCTC-3’
*PRKCB* F	5’-AAATTCACCGCCCGCTTCTT-3’
*PRKCB* R	5’-AAGCAGCAAACTTGGCACTG-3’
*GZMB* F	5’-GGCAGATGCAGACTTTTCCT-3’
*GZMB* R	5’-CTCGTATCAGGAAGCCACCG-3’
ENDOG F	5’-GCCATGGACGACACGTTCTA-3’
ENDOG R	5’-CATTTGCCCACAGCACCAAG-3’
GAPDH F	5’-CGAAGGTGGAGTCAACGGATTT-3’
GAPDH R	5’-ATGGGTGGAATCATATTGGAAC-3’

### 2.14 Statistical analysis

Data analytics was implemented through R software. Dissimilarities between cohorts were assessed via the Wilcoxon rank-sum test, with a p-value< 0.05 indicating remarkable differentiation between the two cohorts. For RT-qPCR analysis, the t-test was employed for statistical comparisons (p< 0.05). Notably, **** represented p< 0.0001, *** represented p< 0.001, ** represented p< 0.01, * represented p< 0.05, and ns represented p > 0.05.

## 3 Results

### 3.1 Biological functions and pathways of DE-MPTDNRGs

**Figure 1:**
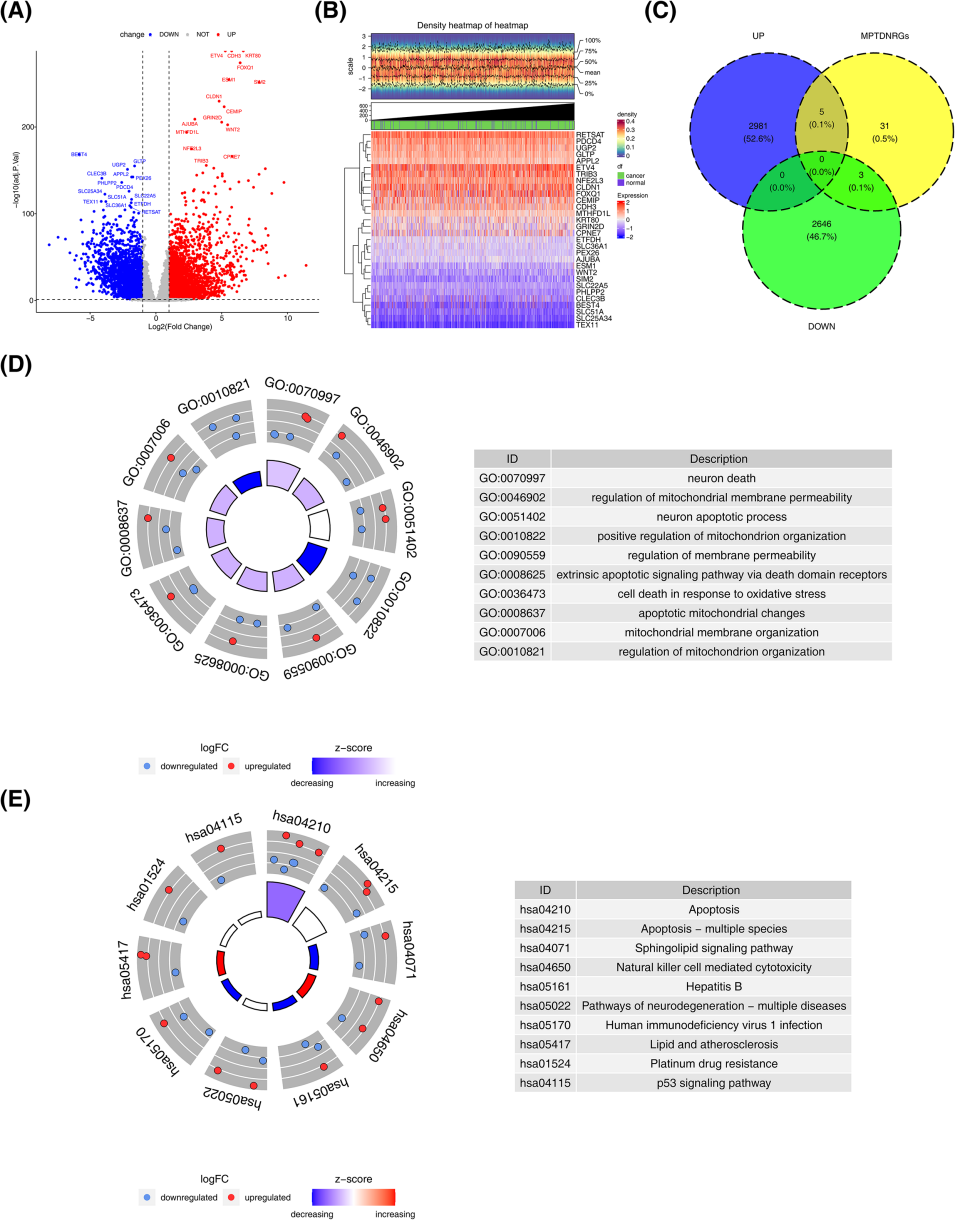
Differential gene screening, identification and enrichment analysis. **(A)** Differential gene volcano map. **(B)** Differential gene density heat map. **(C)** Venn diagram of differentially expressed MPTDNRGs. **(D)** GO enrichment analysis of differentially expressed MPTDNRGs. **(E)** KEGG enrichment analysis of differentially expressed MPTDNRGs in CRC.

There existed 5,635 DEGs identified in TCGA-CRC datasets (tumor vs normal), with 2,986 up-regulated genes and 2,649 down-regulated genes in the normal cohort (**Figures 1A, B**). The DEGs were intersected with MPTDNRGs to attain 8 DE-MPTDNRGs, comprising 5 up-regulated genes and 3 down-regulated genes (**Figure 1C**). To explore the function of DE-MPTDNRGs, enrichment analysis was performed. In results of GO, these genes were mainly concentrated in functions related to mitochondria and neurons, such as neuron death and regulation of mitochondrial membrane permeability, etc. (**Figure 1D**). In addition, these genes were notably involved in Apoptosis and cancer-related pathways, including apoptosis and p53 signaling pathways, etc. (**Figure 1E**).

### 3.2 Functional analysis of prognostic genes

**Figure 2:**
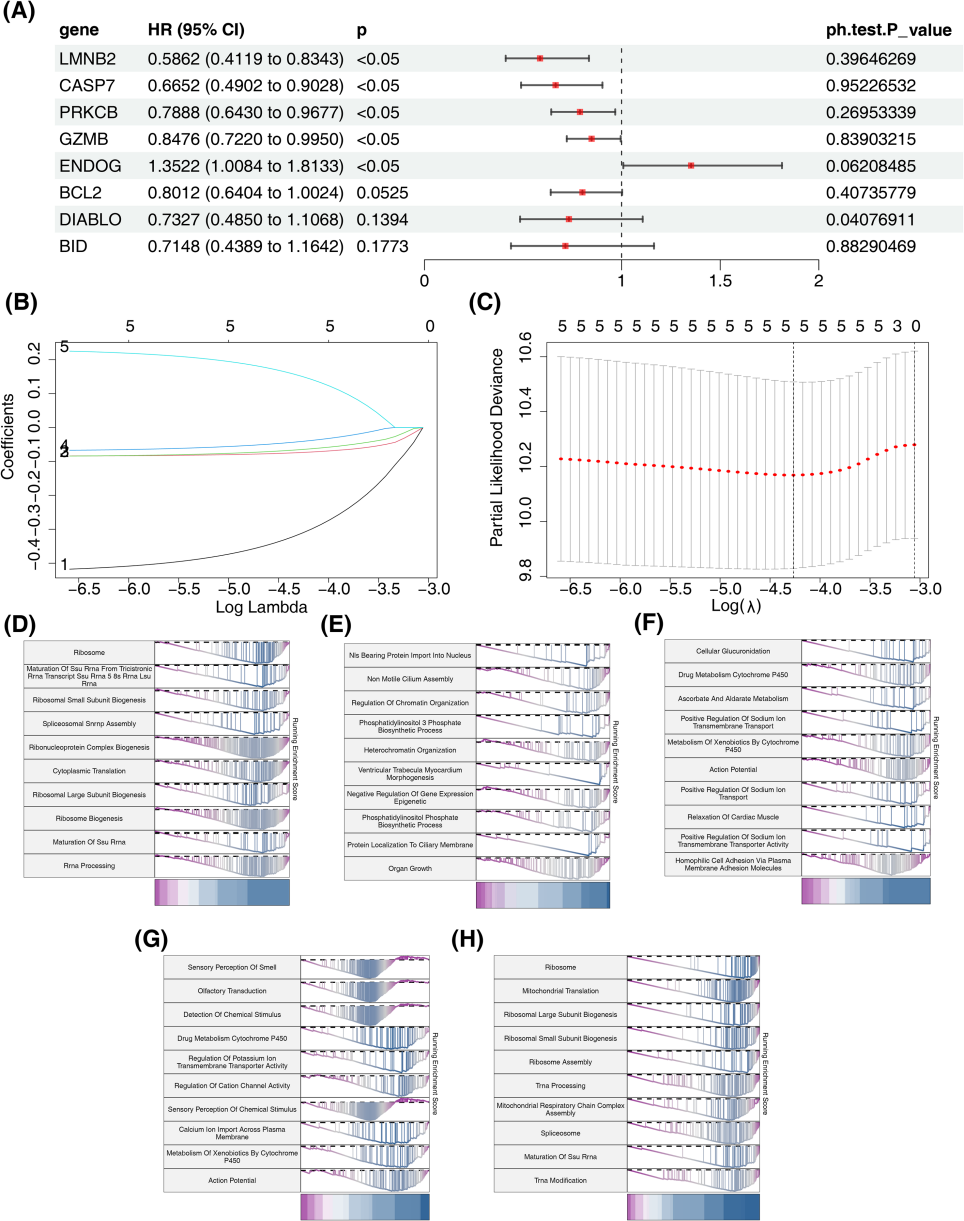
Functional analysis of prognostic genes. **(A)** One-factor cox forest plot of the TCGA-CRC training set. **(B)** Ten-fold cross-validation of tuning parameters in LASSO analysis. **(C)** Lasso coefficient spectrogram. **(D-H)** Single-gene GSEA enrichment analysis of *LMNB2*, *CASP7*, *PRKCB*, *GZMB* and *ENDOG*.

Further, according to 8 DE-MPTDNRGs (*LMNB2*, *CASP7*, *PRKCB*, *GZMB*, *ENDOG*, *BCL2*, *ENDOG*, and *BID*), genes related to the prognosis of CRC were further explored. In the tumor cohort, lower expression levels of *BCL2*, *CASP7*, and *PRKCB* compared to the control cohort were observed, while higher expression levels of *BID*, *ENDOG*, *GZMB*, and *LMNB2* were noted in the tumor cohort compared to the control cohort (**Supplementary Figure 1**). Firstly, univariate Cox regression screened 5 prognostic related genes, all of which passed the PH hypothesis test (**Figure 2A**). Next, these 5 genes were further identified as prognostic genes by LASSO, namely *LMNB2*, *CASP7*, *PRKCB*, *GZMB* and *ENDOG* (**Figures 2B, C**). In addition, we performed survival analysis on the five prognostic genes, and the results showed that patients with low expression levels of *GZMB, LMNB2*, *PRKCB*, and *CASP7* had better survival outcomes, while no significant difference in prognosis was observed for *ENDOG* (**Supplementary Figure 2**). To elucidate the potential function of prognostic genes, enrichment analysis was conducted. The findings revealed that these prognostic genes were similar in functions to a large degree. Several functions might be enriched twice, like ribosome, metabolism of xenobiotics by cytochrome P450, and drug metabolism by cytochrome P450, etc. (**Figures 2D–H**).

### 3.3 Moderately predictive risk model performance

**Figure 3:**
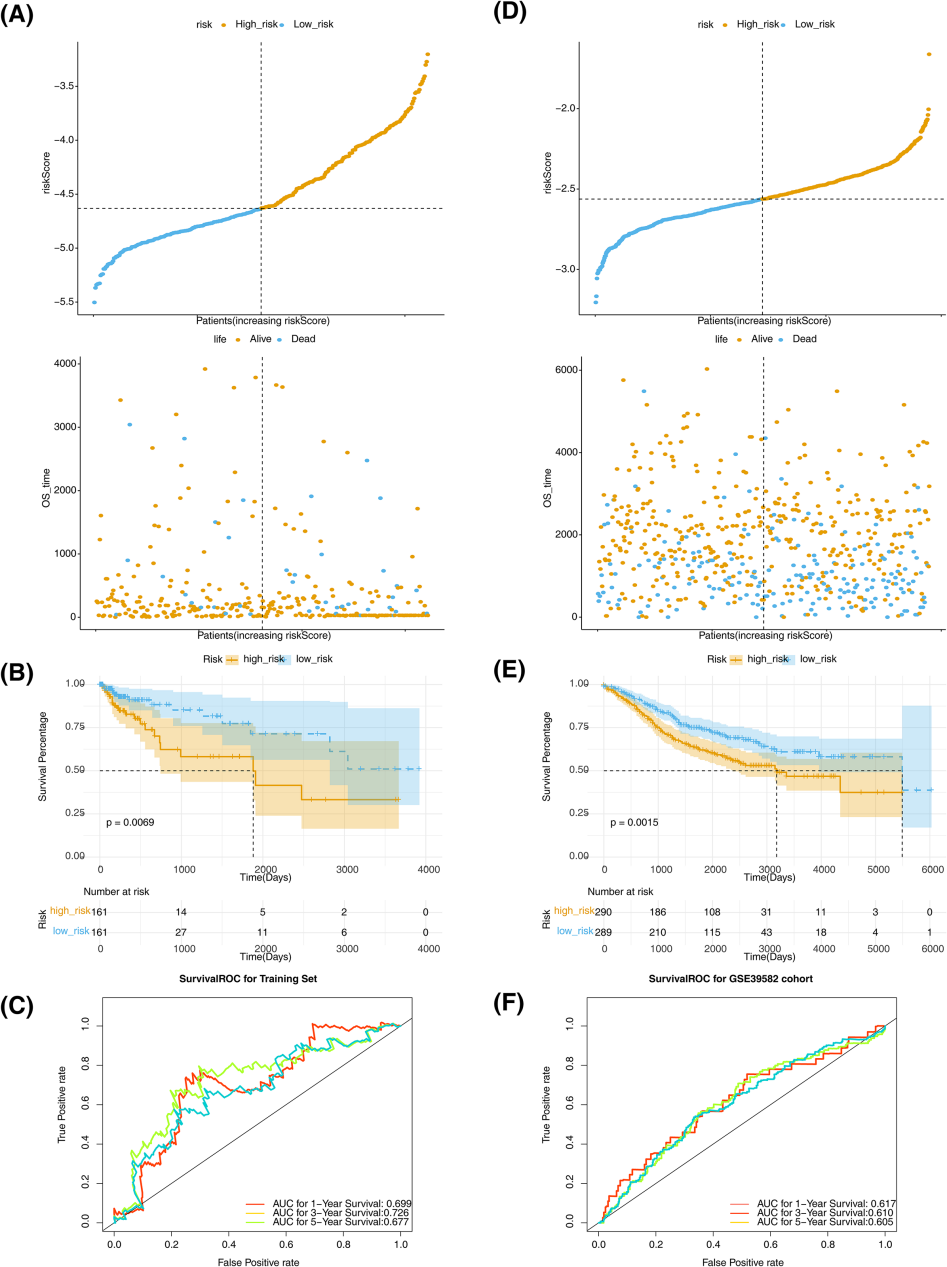
Risk model evaluation and validation. **(A)** Distribution of validation set risk scores (left), distribution of survival states (right). **(B)** Training set K-M curves. **(C)** ROC curves of the training set for years 1, 3, and 5. **(D)** Validation set risk score distribution (left), survival state distribution (right). **(E)** Validation set K-M curve. **(F)** Validation set 1-, 3- and 5-year ROC curves.

The risk score for 5 genes was derived using the formula: riskScore = -0.31130104*LMNB2-0.073557728*CASP7-0.06477476*PRKCB-0.04099177*GZMB + 0.15054822*ENDOG. Afterward, patients in the training dataset were stratified into two risk cohorts using the median risk score (-4.632085) as the cutoff. There was a direct relevance between the increase in risk score and the mortality rate (**Figure 3A**). As proved by the K-M curve analysis, patients in the low-risk cohort had obviously higher survival rates relative to those in the high-risk cohort during the identical period (p = 0.0069) (**Figure 3B**). Additionally, the area under the curve (AUC) values at 1-, 3-, and 5- years were all above 0.6, demonstrating that the risk score provided moderately predictive capability for patients survival (**Figure 3C**). Importantly, similar results were obtained in the GSE39582 validation dataset, highlighting the broad applicability and robustness of the risk model (**Figures 3D–F**).

### 3.4 Prognostic value and predictive capability of independent prognostic factors

**Figure 4:**
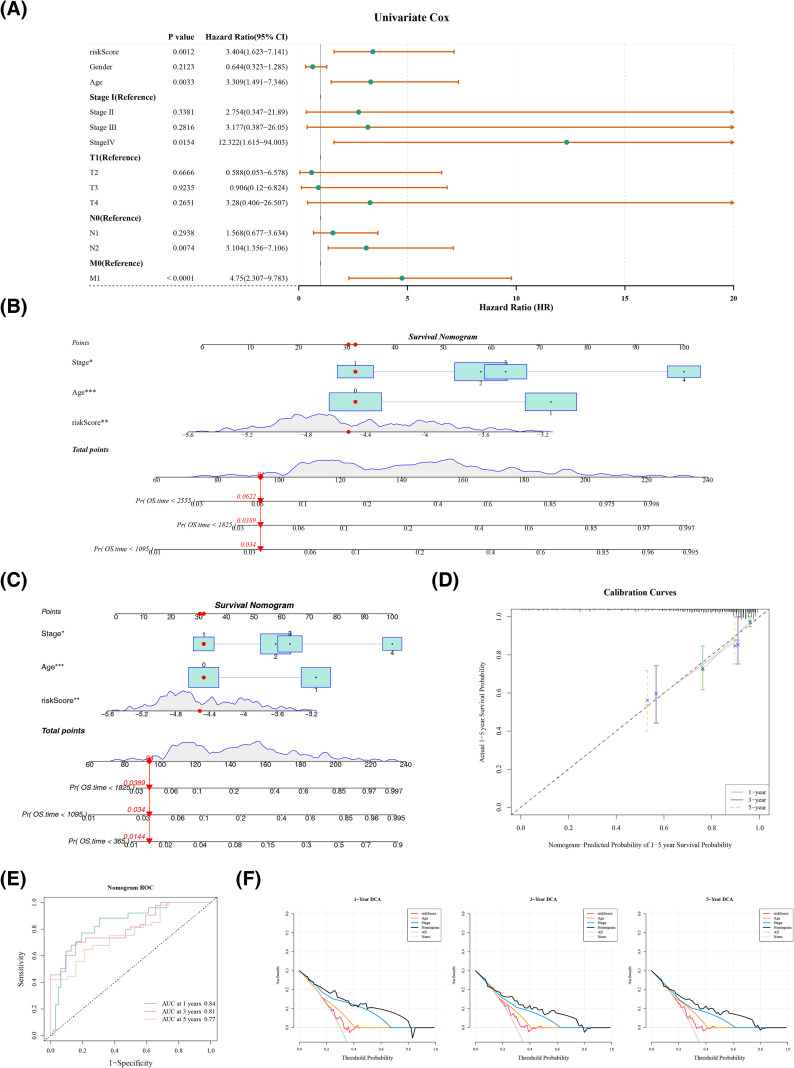
Prognostic value and predictive capability of independent prognostic factors. **(A)** One-factor cox forest plot. **(B)** Multi-factor cox forest plot. **(C)** Line plots predicting 1-, 3-, and 5-year survival probabilities. **(D)** Calibration curves at 1, 3, and 5 years. **(E)** 1-, 3-, and 5-year ROC curves. **(F)** 1-, 3-, and 5-Year DCA Curves of the Nomogram. The x-axis represents the Threshold Probability, which denotes the minimum risk threshold for clinicians to judge the occurrence of adverse outcomes (1-year/3-year/5-year mortality) in patients. The y-axis indicates the Net Benefit, calculated by the formula: “(True Positive Rate × Prevalence) − (False Positive Rate × (1 − Prevalence) × Threshold Probability/(1 − Threshold Probability))”. It directly reflects the additional benefit of using the prediction tool to guide clinical decisions compared with the “no intervention” or “all intervention” strategies under a specific threshold probability, with higher values indicating stronger clinical practical value. The curves in the figure include the DCA curves of the nomogram and independent prognostic factors.

Independent prognostic factors were further screened. The PH assumption test demonstrated that the effects of the risk score and all clinical characteristics on the survival time of colorectal cancer (CRC) patients did not change over time (p > 0.05) (**Supplementary Figure 3A**). Subsequently, univariate Cox analysis illustrated that risk score (HR = 3.404), age (HR = 3.309), N2 stage (HR = 3.104), M1 stage (HR = 4.75), and stage IV (HR = 12.322) were significantly associated with the overall survival (OS) prognosis of patients (p< 0.05) (**Figure 4A**). Due to the extremely small sample size of patients with M stage metastasis (only 1 case), this variable was excluded, and the PH assumption test was re-performed on the aforementioned variables. The results confirmed that their effects on CRC patient survival remained stable (p > 0.05) (**Supplementary Figure 3B**). Multivariate Cox regression deeply retained age (HR = 4.305), risk score (HR = 3.140), and stage IV (HR = 23.231) as IPFs (**Figure 4B**). A nomogram integrating IPFs was formulated to predict the 1-, 3-, and 5-years survival probabilities of CRC patients (**Figure 4C**). The calibration curve revealed that the predicted outcomes for 1-, 3-, and 5- years closely aligned with the diagonal line, indicating a strong predictive capability (**Figure 4D**). Additionally, the AUC values for different years exceeded 0.65, indicating the model’s effectiveness in prediction (**Figure 4E**). Finally, the decision curve analysis (DCA) curve indicated that the clinical utility of the nomogram was superior to that of individual prognostic factors and lay above the extreme curves (“None” and “All”) (**Figure 4F**). In summary, these findings confirm the model’s robust performance.

Furthermore, compared with other published models, the 5-gene prognostic signature in this study exhibited the highest concordance index (Cindex = 0.697) and superior prognostic performance for 1-year, 3-year, and 5-year survival of CRC patients (AUC > 0.7) (**Supplementary Figures 4A, B**).

### 3.5 Mechanisms and pathways of prognostic correlation

**Figure 5:**
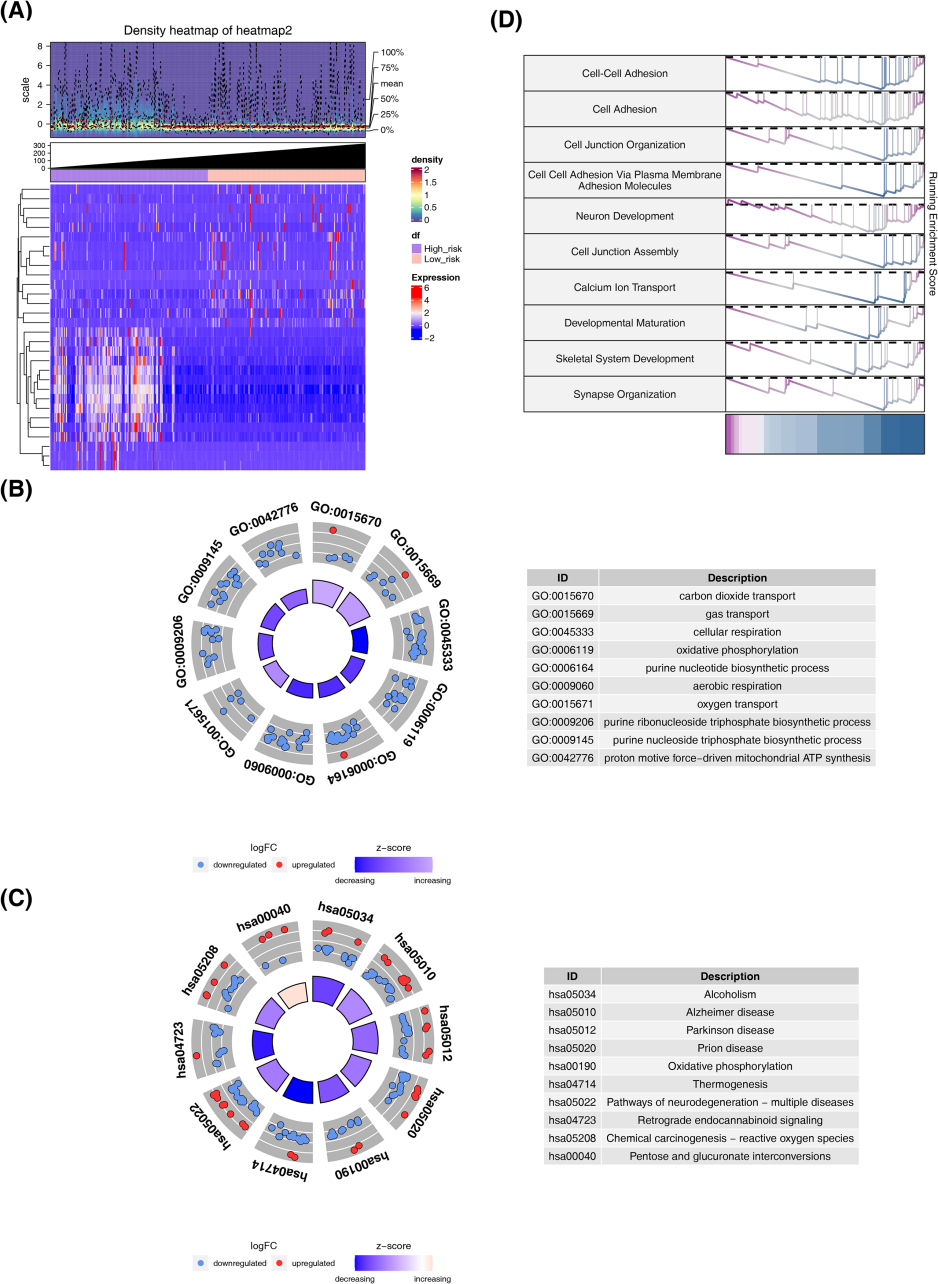
High and low risk group difference analysis and functional enrichment. **(A)** Heat map of genes differing between high and low risk groups. **(B)** GO enrichment results of differential genes in high and low risk groups. **(C)** KEGG enrichment results of differential genes in high and low risk groups. **(D)** Differential gene GSEA enrichment results in high and low risk groups.

There existed 565 DEGs between the two risk cohorts, among which 440 were up-regulated and 125 down-regulated (**Figure 5A**). To further explore the underlying mechanisms of this prognostic relationship, enrichment analysis was implemented (**Figures 5B–D**). The above-mentioned DEGs were mainly enriched in various biological reactions, like oxidative phosphorylation. Moreover, DEGs primarily contained a variety of neurological diseases, such as Alzheimer’s disease and Parkinson’s disease. Moreover, DEGs were also involved in Cell Adhesion and Cell Junction Organization, etc.

### 3.6 A series of immune analyses for different risk cohorts

**Figure 6:**
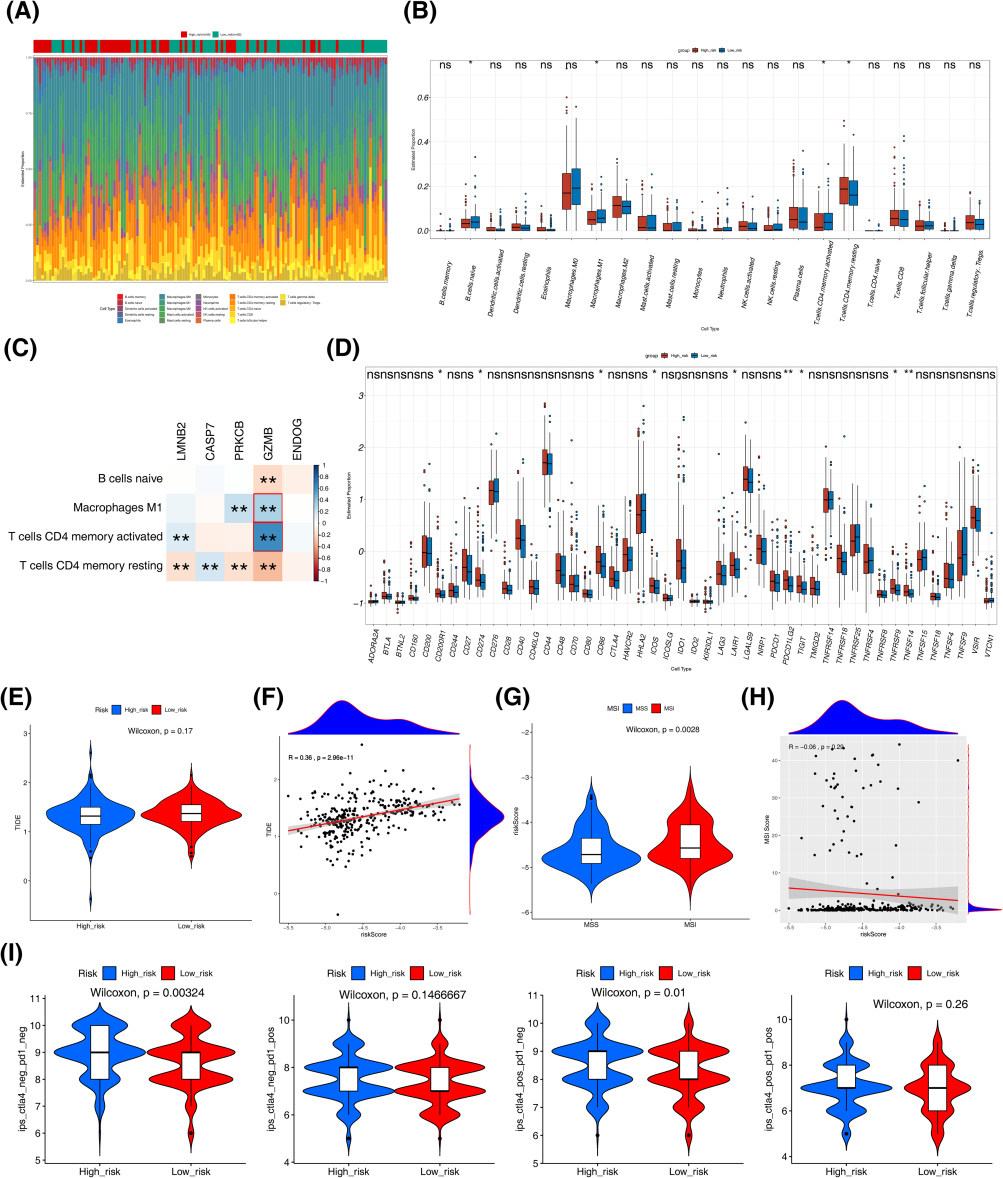
Tumor immune microenvironment analysis. **(A)** Distribution of 22 immune cells in the training set of CRC patients. **(B)** Box plots of differential expression of immune cells between high and low risk groups in the training set. **(C)** Correlation analysis between risk scores and differential immune cells (*p<0.05, **p<0.01), combinations with absolute correlation values >0.3 are marked in red. **(D)** Differential expression levels of 47 immune checkpoints in high and low risk groups (not significant (ns) p>0.05, *p<0.05, **p<0.01). **(E, F)** Differences in TIDE values between high and low risk groups and correlation between risk scores and TIDE values. **(G, H)** Levels of risk scores in MSI and MSS groups and correlation of risk scores with MSI scores. **(I)** Differences in IPS scores between high and low risk groups.

As shown in **Figures 6A**, the proportion of 22 immune cells in 138 patients with p< 0.05 and cell content not all 0 in the training set. In the high-risk cohort, there existed a comparatively higher percentage of resting memory CD4 T cells. Conversely, the proportions of naive B cells, M1 macrophages, and activated memory CD4 T cells were lower (**Figure 6B**). Further, *GZMB* was obviously correlated with 4 different immune cells, and resting memory CD4 T cells was greatly correlated with several other genes except *ENDOG* (**Figure 6C**). In the xCell algorithm, compared with the low-risk cohort, the high-risk cohort exhibited higher infiltration abundances of CD4+ effector memory T cells (CD4+ Tem) and megakaryocyte-erythroid progenitors (MEP) (**Supplementary Figures 5A, B**). In contrast, the high-risk cohort showed lower infiltration abundances of CD4+ memory T cells, CD8+ naive T cells, CD8+ T cells, CD8+ central memory T cells (CD8+ Tcm), mast cells, naive B cells, and T helper 2 cells (Th2 cells) (**Supplementary Figures 5A, B**). Overall, the correlations between these differentially infiltrated immune cells and the prognostic genes were not strong. Mast cells displayed the strongest negative correlation with ENDOG (cor = -0.42, p< 0.001), while CD4+ memory T cells showed the strongest positive correlation with PRKCB (cor = 0.51, p< 0.001) (**Supplementary Figure 5C**). A total of 9 immune checkpoints showed significant differences (p< 0.05), namely CD200R1, CD276, CD86, ICOS, LAIR1, PDCD1LG2, TIGIT, TNFRSF9, TNFRSF14, and their expression was high in the high-risk cohort (**Figure 6D**).

Additionally, no significant difference in TIDE values between two risk cohorts; meanwhile, there was a moderate positive correlation (r = 0.36) observed between the risk score and TIDE values (**Figures 6E, F**). Furthermore, a noteworthy disparity in risk scores was observed between the MSI and MSS cohorts (p = 0.0014), with no significant correlation found between the MSI sensor score and risk score (**Figures 6G, H**). Subsequently, substantial disparities were identified between the two risk cohorts regarding the CTLA4-PD1-score and CTLA4+PD1-score, but not in the other two scores (**Figure 6I**).

### 3.7 Differential drug sensitivity in risk cohorts

**Figure 7:**
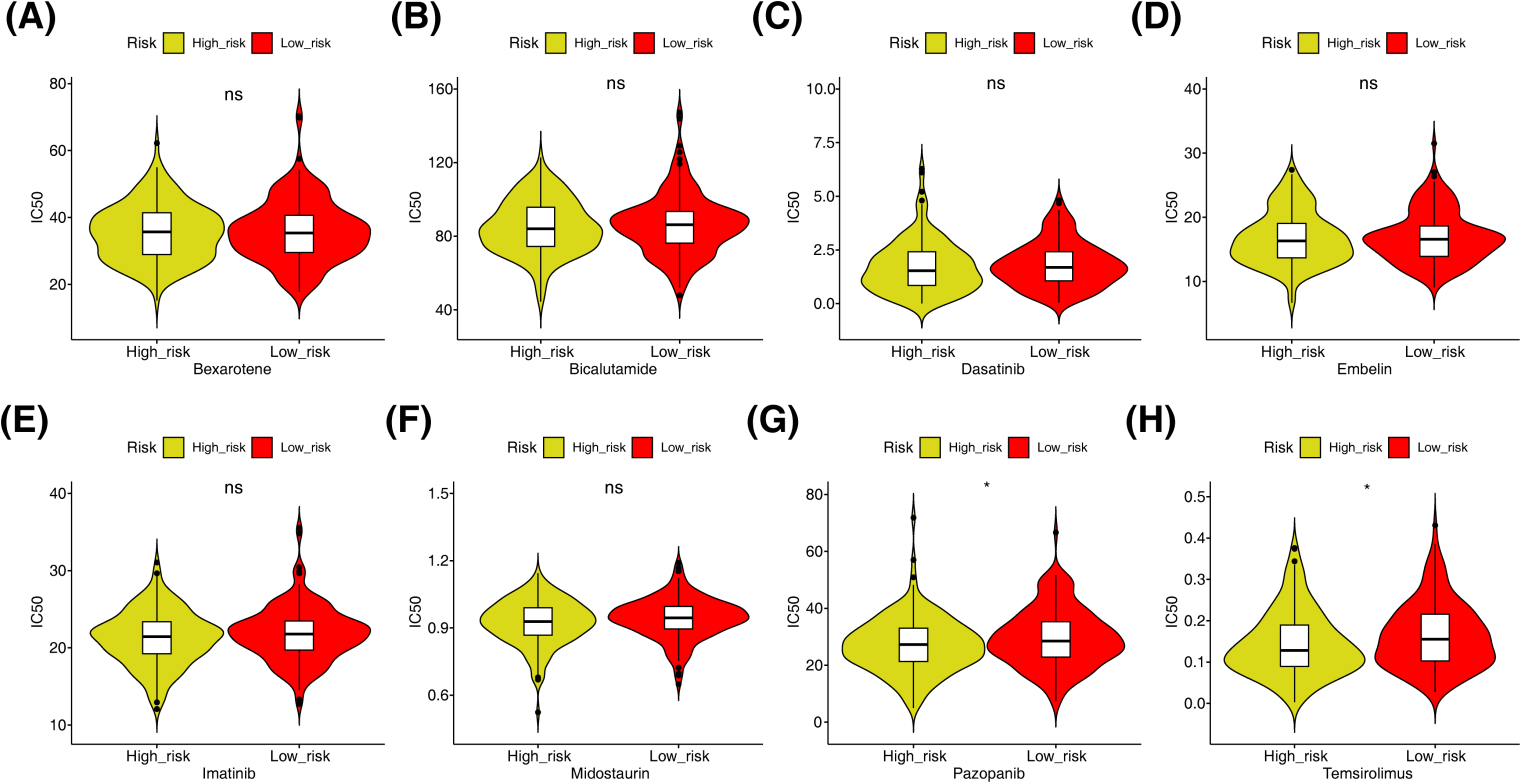
Differences in sensitivity between high and low risk groups for 8 common drugs. **(A–H)** Bexarotene, bicalutamide, dasatinib, embelinin, imatinib, midostaurin, pazopanib and temsirolimus (not significant (ns) p>0.05, *p<0.05).

In addition, the sensitivity of different risk cohorts to therapeutic drugs was further compared. The results revealed that among the 8 commonly used chemotherapy and molecular targeted drugs, Pazopanib and Temsirolimus had higher IC_50_ values in the low-risk cohort (**Figures 7A–H**).

### 3.8 Results of single-cell analysis

**Figure 8:**
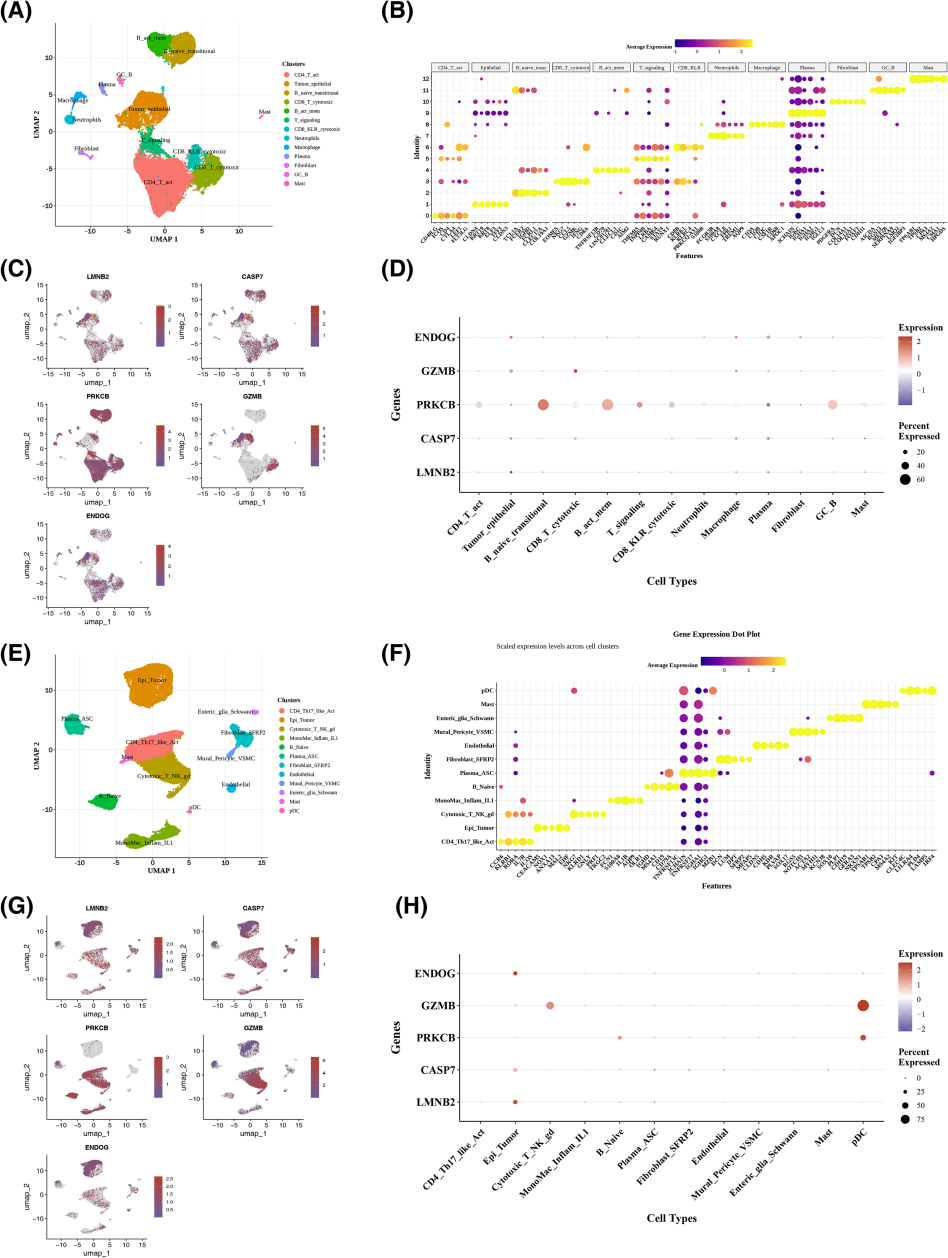
Expression of prognostic genes across different cell types in single-cell datasets. **(A)** Annotated Uniform Manifold Approximation and Projection (UMAP) plot of the GSE282542 dataset, where different colors correspond to distinct cell types. **(B)** Bubble plot showing highly expressed marker genes across cell types in the GSE282542 dataset. The x-axis represents marker genes, the y-axis represents cell types, the color indicates the standardized expression level, and the bubble size denotes the proportion of the marker gene within each cell type. **(C)** UMAP plot illustrating the expression of prognostic genes in cells from the GSE282542 dataset. **(E)** Bubble plot showing the proportion of prognostic genes across cell types in the GSE282542 dataset. The color represents the average expression level, and the bubble size indicates the expression proportion. **(F-H)** Annotated UMAP plot of cells, bubble plot of marker gene expression, UMAP plot of prognostic gene expression, and bubble plot of prognostic gene expression in the GSE132465 dataset, respectively.

In the GSE282542 dataset, following stringent QC, 38,037 high-quality cells and 29,655 genes were preserved for further analysis (**Supplementary Figures 6A, B**). The top 2,000 highly variable genes were selected for subsequent analysis, and 10 of the most variable genes, encompassing IGHA2, IGKC, and IGHG1, were emphasized (**Supplementary Figure 7A**). Thereafter, the PCA was performed on these genes for dimensionality reduction, and the top 20 principal components, based on statistical significance (P< 0.05), were selected (**Supplementary Figures 7B–D**). A total of 13 cell populations were ultimately identified (**Supplementary Figure 7E**). Cell annotation was performed via marker gene analysis, identifying 13 distinct cell types: CD4+ activated T cells, tumor epithelial cells, naive/transitional B cells, CD8+ cytotoxic T cells, activated memory B cells, signaling-competent T cells, CD8+ KLR+ cytotoxic T cells, neutrophils, macrophages, plasma cells, fibroblasts, germinal center B cells, and mast cells (**Table 2**; **Figure 8A**). Marker genes exhibited highly specific expression in these cell types, confirming the reliability of the annotations (**Figure 8B**). Additionally, biomarkers were expressed in multiple cell types: specifically, *LMNB2*, *ENDOG*, and *CASP7* were expressed in tumor epithelial cells, CD4+ activated T cells, CD8+ cytotoxic T cells, and other cell types; *PRKCB* exhibited relatively high expression in CD4+ activated T cells, activated memory B cells, naive/transitional B cells, and other cell types; while *GZMB* showed relatively high expression in tumor epithelial cells and other relevant cell types (**Figures 8C, D**).

**Table 2:** The annotation of marker genes in all cell samples.

Cell type	Cluster	Cell numbers	Marker gene
Epithelial cell	0	5759	–
T helper 2(Th2) cell	1	4685	SCGB3A1, PMCH
B cell	2	3175	IGHV3-20
Monocyte	3	1892	HLA-DRB5
Gamma delta T cell	4	1793	TRGV9, TRGV4, TRDV2
Oogenesis phase fetal germ cell	5	1621	TAFA1, ADSSa
Mitotic arrest phase fetal germ cell	6	1264	UTS2B
NK cell	7	1235	GZMA, CCL5, NKG7
Plasma cell	8	1112	IGHG3
SLC16A7+ cell	9	1045	MOGAT3
Macrophage	10	638	S100A8, S100A9, MPEG1, S100A12
Mast cell	11	491	CPA3, HPGDS, TPSB2, GATA2, TPSAB1
Stem cell	12	76	MSI1

In the GSE282542 dataset, cells were clustered into 12 distinct cell clusters (**Supplementary Figures 8A–F**), and 12 cell types were annotated: Tumor Epithelial cell (Epi_Tumor), Plasma cell-Antibody-Secreting Cell (Plasma_ASC), Enteric glial cell-Schwann cell (Enteric_glia_Schwann), Activated CD4+ T cell-Th17-like T cell (CD4_Th17_like_Act), Fibroblast-SFRP2+ Fibroblast (Fibroblast_SFRP2), Cytotoxic T cell-Natural Killer cell-Gamma delta T cell (Cytotoxic_T_NK_gd), Inflammatory Monocyte-Macrophage-IL1+ Cell (MonoMac_inflam_IL1), Naive B cell (B_Naive), Mast cell (Mast), Mural cell-Pericyte-Vascular Smooth Muscle Cell (Mural_Pericyte_VSMC), Endothelial cell (Endothelial), and plasmacytoid dendritic cell (pDC) (**Figure 8E**). Bubble plots showed the highly expressed marker genes in each cell type (**Figure 8F**). The prognostic genes ENDOG, CASP7, and LMNB2 were highly expressed in tumor epithelial cells; GZMB was highly expressed in Cytotoxic_T_NK_gd and pDC cells; and PRKCB was highly expressed in B cells and pDC cells (**Figures 8G, H**). In summary, the expression patterns of the prognostic genes across cell types were generally consistent between the two single-cell datasets.

### 3.9 Validation of prognostic genes in clinical samples

**Figure 9:**
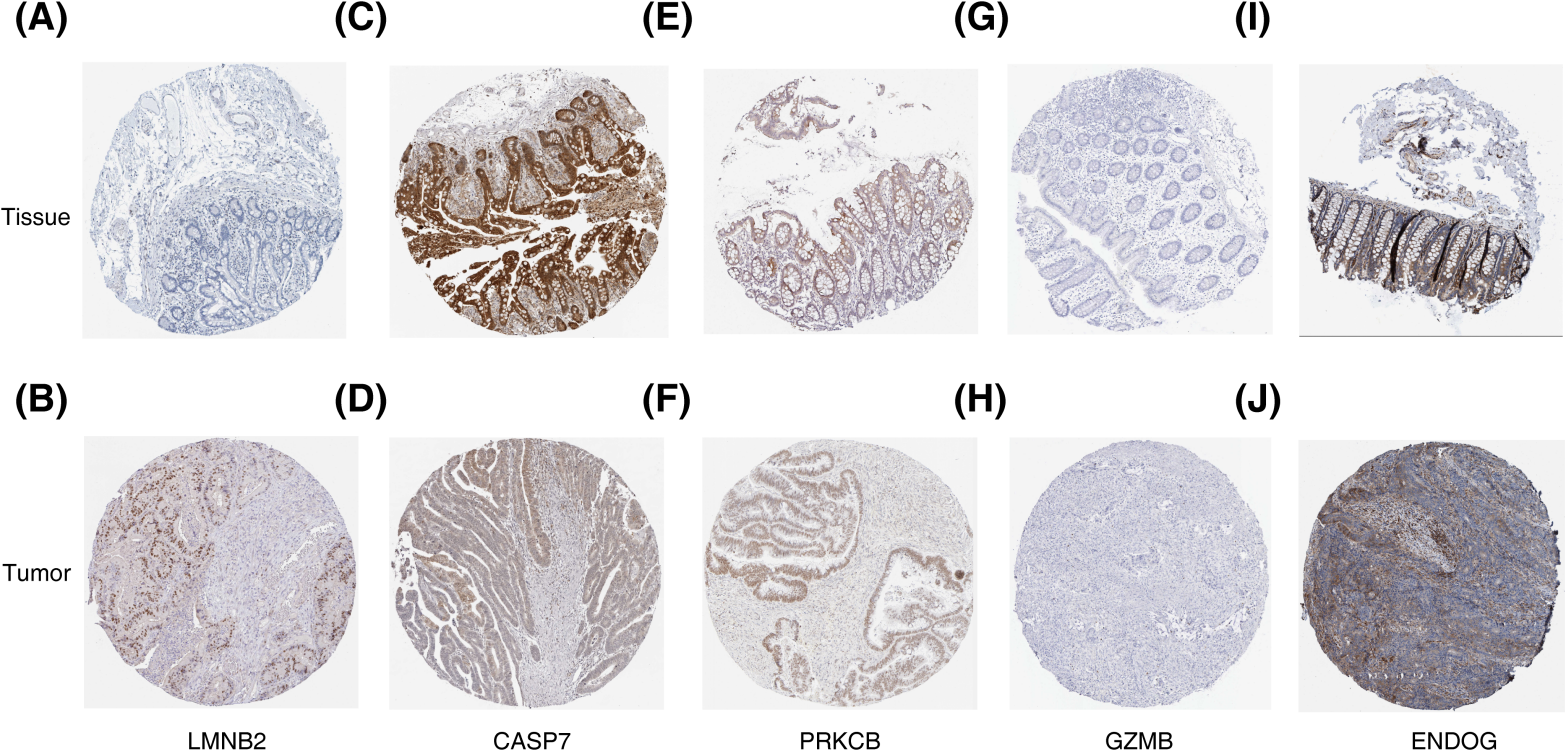
Comparison of the protein expression of prognostic genes in normal colon tissues and colorectal cancer (CRC) tissues. **(A)** The protein expression pattern of *LMNB2* in normal colon tissue. **(B)** the expression pattern of *LMNB2* in colorectal cancer tissue. **(C)** The protein expression of *CASP7* in normal colon tissue. **(D)** the expression of *CASP7* in colorectal cancer tissue. **(E)** The expression of *PRKCB* in normal colon tissue. **(F)** the expression of *PRKCB* in colorectal cancer tissue. **(G)** The expression of *GZMB* protein in normal colon tissue. **(H)** the expression of *GZMB* protein in colorectal cancer tissue. **(I)** The expression of *ENDOG* protein in normal colon tissue. **(J)** the expression of *ENDOG* protein in colorectal cancer tissue.

**Figure 10:**
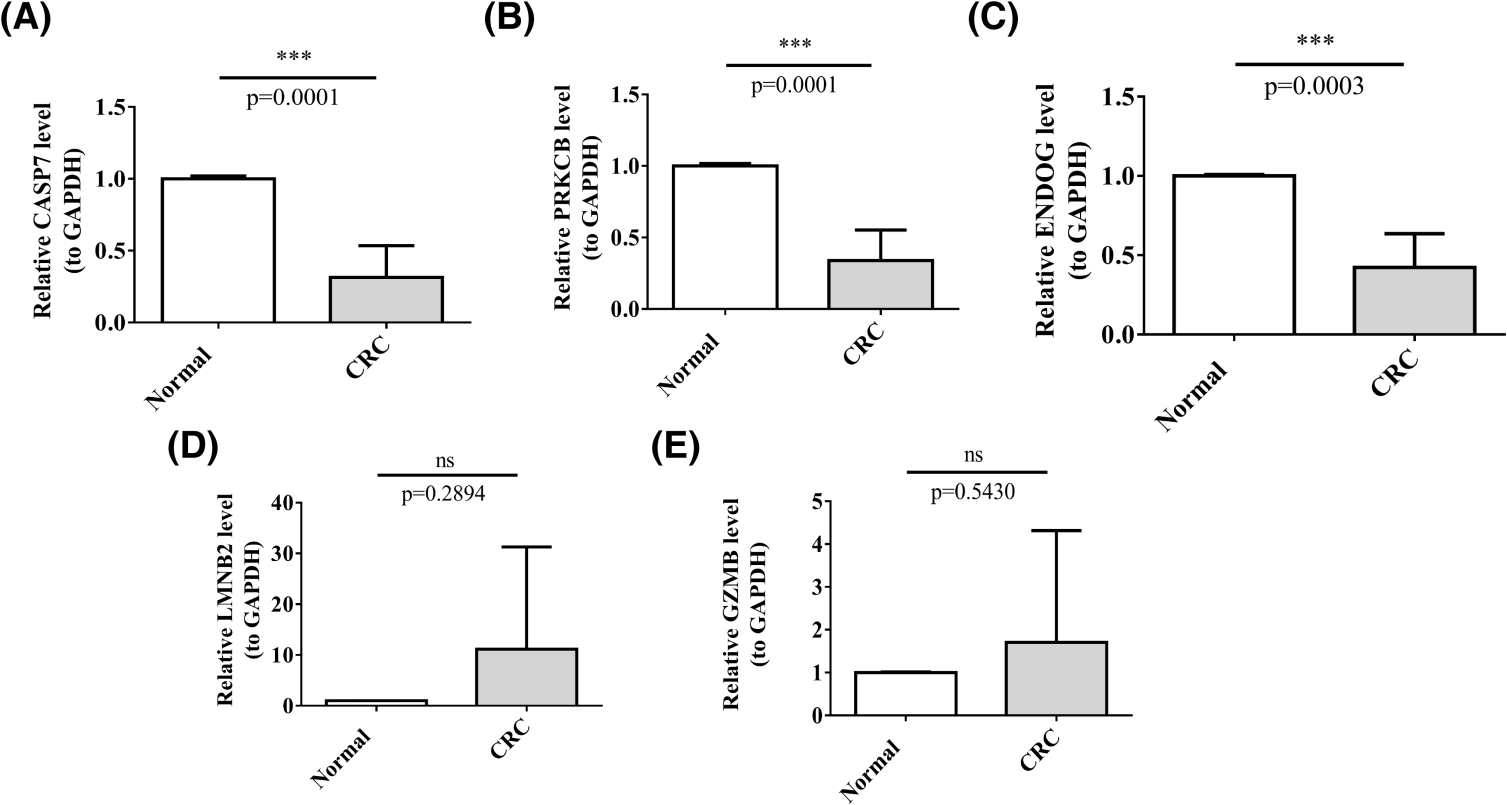
Validation of prognostic genes in clinical samples. **(A–E)** Expression of *CASP7*, *PRKCB*, *ENDOG*, *LMNB2* and *GZMB*. (not significant (ns) p>0.05, ***p<0.001).

To construe the prognostic genes at the protein level, we employed the online database of the Human Protein Atlas (HPA). We construed the protein expression levels of the prognostic genes in CRC tissue specimens. Findings showed that, relative to normal colon tissue samples, *LMNB2* was highly expressed in CRC tissue samples; *CASP7* and *PRKCB* possessed lower expressions in CRC tissue specimens; *GZMB* had low expressions in both normal colon tissue and CRC tissue samples; and *ENDOG* possessed the elevated expressions in both healthy colon tissue samples and CRC tissue samples (**Figures 9A–J**). Clinical samples were collected to examine the expression of prognostic genes in CRC tissues and controls. RT-qPCR results implied that, in the CRC cohort, the mRNA expression levels of *CASP7*, *PRKCB*, and *ENDOG* were prominently reduced, relative to the control cohort. Nonetheless, no statistically significant differences in mRNA expression levels of *LMNB2* and *GZMB* between the two cohorts were found (**Figures 10A–E**).

## 4 Discussion

CRC represents a major global health concern, standing as the second in terms of mortality and third in incidence among all cancers worldwide (32). The debilitating symptoms of CRC and the side effects of its treatment severely compromise patients’ quality of life (33). Despite advances in screening and therapy, the prognosis for CRC remains poor, particularly in advanced stages, underscoring the urgent need for improved diagnostic and prognostic tools. In the area of CRC, the focus has primarily been on the importance of mitochondrial dynamics in cancer cell metabolism and survival. Wu et al. highlighted the influence of mitochondrial fusion-fission dynamics on CRC biology (34), suggesting a dual role in CRC development. Our study has developed a potentially useful risk model in view of 5 DE-MPTDNRGs, including *LMNB2*, *CASP7*, *PRKCB*, *ENDOG*, and *GZMB*, which effectively stratified patients into low- and high-risk cohorts with remarkably different mortality rates and survival outcomes. The risk model exhibited good predictive capability and was further validated in an independent dataset, demonstrating its universal applicability. Moreover, functional and immune analyses revealed potential mechanisms and therapeutic implications of DE-MPTDNRGs in CRC.

CASP7 functions as a regulatory hinge between MPTDN and apoptosis in colorectal cancer. Pan-cancer transcriptomes uniformly show lower CASP7 mRNA in tumors versus matched mucosa (35), and reduced expression correlates with a 13% decrease in 5-year survival); this aligns with our findings. At single-cell resolution we mapped CASP7 to malignant epithelia and CD8^+^ cytotoxic T cells, revealing a lineage-restricted control axis. Post-transcriptionally, TRIM25 docks on the CASP7 3′-UTR and recruits hnRNPH1 to accelerate mRNA decay, blunting chemotherapy-induced apoptosis (35). Conversely, SREBP1 blockade restores CASP7 and potentiates gemcitabine lethality (36), whereas miR-519a-3p directly targets CASP7 and dampens death signaling (37). In ischemia–reperfusion models the MPT couples energy status to cell fate: glycolytic ATP rebound redirects cells from necrosis to caspase-3-mediated apoptosis, a switch abolished by cyclosporin A or caspase-3 inhibitors (38). Together, these data establish CASP7 as the cross-point where MPTDN and apoptotic circuits merge to govern chemosensitivity and prognosis in CRC.

Consistent with our findings, PRKCB (Protein Kinase C Beta) is transcriptionally down-regulated in CRC tissues, and its higher expression is significantly associated with better overall survival (39). Single-cell RNA-seq data implied a dual role in sculpting the tumor immune micro-environment and maintaining cancer stem-cell properties. A similar inverse correlation between PRKCB promoter hyper-methylation and reduced mRNA abundance has been documented in gastric cancer, where methylation of cg08406370 and cg00735962 CpG islands silences PRKCB and predicts poor prognosis (40). Comparable epigenetic repression may therefore underlie the low PRKCB levels observed in our CRC cohort. Extending beyond gastrointestinal malignancies, a large retrospective NSCLC study demonstrated that robust PRKCB expression independently confers favorable prognosis (HR = 0.47, P< 0.001) and negatively correlates with advanced TNM stage (41). At the mitochondiral metabolic level, recent work in ischemia-resistant hippocampal neurons has positioned PKCβII as a critical regulator of mitochondrial glutaminase activity and subsequent anaplerotic flux through the TCA cycle (42); whether PRKCB-mediated phosphorylation events similarly modulate mitochondrial substrate utilization—glutamine versus pyruvate oxidation—in CRC stem or immune cells remains an attractive, yet untested, mechanism that could link its prognostic impact to metabolic plasticity.

ENDOG is an endonuclease situated in the mitochondrial intermembrane space, is instrumental in orchestrating DNA fragmentation and apoptosis through its translocation to the nucleus, and it also modulates mitochondrial genome cleavage by relocating to the mitochondrial matrix (43). In ischemic cardiomyocytes, Bnip3-induced mitochondrial permeability transition drives EndoG release from mitochondria and subsequent caspase-independent DNA fragmentation, establishing a direct mitochondrial-nuclear death axis that operates independently of caspases (44). Studies have indicated that *ENDOG* can be phosphorylated by GSK3B, which strengthens its interaction with YWHAG. This process results in the release of TSC2 and PIK3C3 from YWHAG, subsequent suppression of the MTOR signaling pathway, and initiation of autophagy. Additionally, the endonuclease activity of *ENDOG* is vital for activating the DNA damage response and thus inducing autophagy (43, 45). Previous studies have revealed that compounds like amentoflavone (46) and the LL-37-derived peptide FK-16 (47) can induce apoptosis, possibly through mechanisms involving *ENDOG*. Additionally, increased expression of *ENDOG* in cancers (48) further supports its potential role in cancer apoptosis. Research has also found that *ENDOG* deficiency or silencing inhibits proliferation in certain cancer cell lines (e.g., endometrial tumors and glioblastomas), especially when PTEN is missing or p-AKT is highly expressed in these tumor cells, suggesting that low *ENDOG* expression may limit tumor growth (43). However, studies on colorectal cancer indicate that substituted diaryl diselenides show complex cytotoxic and apoptotic effects on colon adenocarcinoma cells (49), suggesting that *ENDOG*’s role in colorectal cancer may not be entirely beneficial and requires further study. Although TCGA data show ENDOG up-regulation in CRC, our RT-qPCR cohort revealed significant down-regulation, and survival analysis detected no stratification between high- and low-ENDOG groups, highlighting the need for expanded samples and functional validation to resolve this discrepancy.

LMNB2 (Lamin B2) is an integral nuclear-membrane protein that orchestrates chromatin remodeling and the rupture–re-assembly of the nuclear envelope during mitosis, thereby governing eukaryotic cell proliferation and tumorigenesis (50). It can silence the tumor-suppressor gene p21, creating a cellular environment conducive to colorectal cancer (CRC) advancement (50). In hepatocellular carcinoma, high LMNB2 expression is tightly linked to immune-evasive signatures within the tumor microenvironment, implying a broader immunomodulatory role (51). Recent work shows that LMNB2 fosters cancer-cell stemness and the Warburg effect via activation of the p38 MAPK cascade—a signaling node that cross-talks with mitochondrial permeability transition and oxidative-stress responses—thus providing an indirect but biologically plausible connection between LMNB2 and mitochondrial functionality (52). Additionally, a regulatory circuitry involving long non-coding RNAs and microRNAs (e.g., miR-326) fine-tunes LMNB2 abundance, influencing CRC progression (53). Consistent with these findings, our analyses identified significant LMNB2 up-regulation in CRC tissues, and high expression was associated with shorter survival.

Granzyme B (*GZMB*), a key constituent of cytotoxic granules in natural killer (NK) cells and cytotoxic T lymphocytes (CTLs), is crucial for the immune system’s defense against cancer (54). Overexpression of *GZMB* is often linked to poor prognosis in colorectal cancer (CRC) patients and associated with more aggressive CRC phenotypes (55). However, some studies suggest that high *GZMB* expression may improve survival by enhancing antitumor immune responses (56).This dual role indicates that *GZMB*’s prognostic impact may depend on tumor stage, molecular features, and the immune microenvironment (57). Additionally, low *GZMB* expression may be related to immune evasion in colon cancer, influencing NK cell cytotoxicity and cytokine signaling pathways, therefore promoting tumor progression (43). In CRC, mutations in genes like KRAS and BRAF are well-documented. KRAS mutations are closely related to cancer cell proliferation, survival, and metastasis, and can lead to treatment resistance (43, 58, 59). In right-sided colon cancer, the BRAF (V600E) mutation is often associated with highly aggressive tumors and poor prognosis (60). Similar to KRAS mutations, BRAF mutations also frequently result in treatment resistance, particularly to anti-EGFR monoclonal antibody therapy (61, 62).

Unlike KRAS and BRAF mutations, *GZMB* is mainly related to immune responses and has a dual role in the tumor microenvironment. For one thing, the elevated *GZMB* expression may promote tumor immune evasion and progression. For another, it may enhance antitumor immune responses. Therefore, *GZMB*’s function is closely related to the tumor immune environment and molecular characteristics. Although *GZMB* overexpression may serve as a negative prognostic biomarker in CRC, precisely explaining its clinical importance needs a thorough investigation into its interactions with the immune microenvironment.

We conducted enrichment analysis to detect the possible underlying mechanism of MPTDNRGs in CRC. Pathways between different risk groups were enriched in biological processes such as oxidative phosphorylation, which is pivotal for cellular energy metabolism and has been implicated in cancer progression and chemoresistance (63). Prior research unveiled that isobavachalcone induced non-apoptotic necrosis among cancer cells by triggering mitochondrial permeability transition, oxidative stress, and the opening of the mitochondrial permeability transition pore, bringing about mitochondrial unbalance and finally cell death (64). As claimed by Zhao et al., NPC-26, a new mitochondrion-interfering compound, triggers CRC cell death by activating AMP-activated protein kinase (AMPK) signaling, which is linked to mitochondrial permeability transition pore (mPTP) opening and reactive oxygen species (ROS) production (65). Furthermore, the enrichment in neurodegenerative disease pathways is consistent with previous research (7). Although these pathways are traditionally associated with neurological disorders, emerging evidence suggests that the molecular mechanisms involved, like protein misfolding and mitochondrial dysfunction, may also play roles in cancer pathogenesis and resistance to therapy (66). Such intersection of neurodegenerative and oncogenic pathways could provide novel insights into CRC biology and identify potential biomarkers for disease progression and therapeutic response.

This risk model not only achieved prognostic stratification but also revealed distinctly different tumor immune microenvironments underlying the outcomes. The high-risk group, associated with unfavorable survival, featured a dysfunctional tumor immune microenvironment (TIME): characterized by accumulation of immunosuppressive cells (elevated resting memory CD4 T cells) (67), reduction of anti-tumor effector cells (decreased naive B cells, M1 macrophages, and activated memory CD4 T cells), and widespread upregulation of immune checkpoint molecules on both tumor and immune cells (such as elevated PDCD1LG2/PD-L2). These coordinated changes collectively indicated an adaptive immune resistance state (68, 69). Notably, the alteration in M1 macrophage infiltration was statistically significant only in the CIBERSORT algorithm but not in the xCell algorithm. Nevertheless, M1 macrophages still exhibited a higher proportion in the low-risk group, which may be attributed to sample heterogeneity and functional exhaustion of immune cells. In conjunction with the immunosuppressive tumor microenvironment observed in the high-risk group, the reduced abundance or impaired function of M1 macrophages likely contributes to enhanced tumor immune evasion, consistent with the unfavorable prognosis observed in high-risk patients. Additionally, the higher proportion of MSS patients in the high-risk group aligned with the typically lower immunogenicity and reduced sensitivity to immunotherapy characteristic of MSS tumors, further reinforcing this group’s “cold” or suppressed immune phenotype (70). Interestingly, while the TIDE score did not significantly differentiate between groups, the risk score itself positively correlated with TIDE, and the high-risk group showed distinct patterns in IPS predicting differential responses to combination immunotherapy (anti-CTLA-4/PD-1). This suggested that the gene signatures captured by our risk model were intrinsically linked to tumor immune evasion mechanisms. Consequently, the poor prognosis in high-risk patients likely resulted from a microenvironment unfavorable for effective anti-tumor immune responses, collectively constructed by MSS background, suppressive immune cell infiltration, and checkpoint upregulation (71). Correspondingly, the low-risk group exhibited favorable immunological characteristics, offering valuable insights for developing personalized immunotherapy strategies in colorectal cancer (CRC). Notably, the high-risk group demonstrated higher predicted sensitivity to Pazopanib. Although currently approved for renal cell carcinoma and advanced soft tissue sarcoma (72), this agent has shown potential in CRC by inducing tumor regression when combined with the FOLFOX regimen (73), and its safety and tolerability have been established in multiple clinical studies (74, 75). We hypothesize that the therapeutic efficacy of Pazopanib may be mediated by the prognostic genes identified in this study. However, it is important to emphasize that this recommendation constitutes an *in silico* prediction rather than a definitive therapeutic guideline. Consequently, its clinical utility requires rigorous validation through functional experiments (e.g., cell lines, organoids, or PDX models) and further clinical investigation.

Our study has several limitations. First, the RT-qPCR validation was constrained by a small sample size. Owing to the restricted sample-use clause in the Ethics Review, we were unable to obtain additional clinical specimens across centers; consequently, the largest feasible cohort was analyzed under the current ethical framework. Although these data provided an initial biological anchor for the in-silico findings, the statistical power is inherently limited, and the observed expression trends require cautious interpretation. Therefore, prior to clinical application, multi-level validation (at both mRNA and protein levels) with an expanded sample size is required. Furthermore, the generalizability of the prognostic gene signature and its potential as a biomarker need to be rigorously validated in large-scale, multi-center prospective cohorts. Second, the study remains predominantly computational; mechanistic insights into how the hub genes modulate MPTDN await functional validation. We propose prioritized experiments—including CRISPR knockout/rescue in CRC organoids, mitochondrial stress tests with high-resolution respirometry, and patient-derived xenograft (PDX) models—to dissect gene-specific effects on oxidative phosphorylation and MPTP opening. Third, drug-sensitivity predictions for pazopanib and temsirolimus were inferred from in-silico IC50 profiling; these findings need prospective verification in 3-D culture systems and PDX models to rule out false-positive biomarker associations. Finally, the current risk model explained a moderate proportion of survival variance; integrating Consensus Molecular Subtypes (CMS) and Immunoscore variables could increment model discrimination and calibration, but this strategy must be formally evaluated through nested cross-validation in external cohorts.

## 5 Conclusions

This study successfully identified differentially expressed genes associated with mitochondrial permeability transition in CRC and build a strong prognostic risk model. Functional enrichment analysis revealed significant involvement of these genes in various biological processes and signaling pathways. The risk model demonstrated strong predictive performance for patient survival, and its independent prognostic value was confirmed. Furthermore, immune analysis and drug sensitivity assessments highlighted significant differences between high- and low-risk groups, providing insights into potential therapeutic strategies. These findings underscore the potential of mitochondrial permeability transition-related genes as biomarkers and therapeutic targets in CRC. Future research should focus on validating these results through experimental and clinical studies to fully realize their potential in improving patient outcomes.

## Data Availability

The data presented in the study are deposited in the figshare repository, accession number 10.6084/m9.figshare.31422392.

## Supplementary material

Supplementary Figure 1 Expression profiles of 8 de-mptdnrgs.

Supplementary Figure 2 Survival curves of the five prognostic genes.

Supplementary Figure 3 Proportional Hazards (PH) Assumption Test Results for Independent Prognostic Analysis. **(A)** PH assumption test results of univariate Cox regression analysis for the risk score and clinical factors. The x-axis (Time) represents the survival time of patients. The y-axis (Beta (t) for riskScore) denotes the time-dependent changes in the regression coefficient corresponding to the risk score, reflecting the temporal dependency of the risk score’s impact intensity on survival risk. The regression coefficient curve in the figure shows no obvious upward or downward trend over the entire follow-up period and fluctuates around the value of 0, which intuitively confirms that the risk effect of the risk score does not change over time, further supporting the statistical conclusion that the proportional hazards assumption is satisfied. The proportional hazards assumption is considered to be met when the P-value is greater than 0.05. **(B)** PH assumption test of multivariate Cox regression analysis for the factors identified by univariate Cox regression analysis.

Supplementary Figure 4 Comparison of the prognostic model with published prognostic models. **(A)** Comparative analysis results of prognostic performance between the MPTDN-5 model (5 prognostic genes) constructed in this study and the published models. Rows include the prognostic gene model established in this study and four cell death-related subtypes, namely mitophagy, cuproptosis, necroptosis, and pyroptosis. Columns consist of five prognostic performance evaluation metrics, namely Concordance Index (Cindex), 1-year area under the curve (AUC_1y), 3-year area under the curve (AUC_3y), and 5-year area under the curve (AUC_5y). The numbers in the table are raw performance values, and the color of each column corresponds to the z-score normalized values. Published refers to previously reported prognostic models of the same category. **(B)** Time-dependent receiver operating characteristic (ROC) curves showing the performance of the MPTDN-5 model in predicting 1-year, 3-year, and 5-year survival outcomes of colorectal cancer (CRC) patients across four cell death-related subtypes (cuproptosis, mitophagy, pyroptosis, necroptosis). The x-axis (1−Specificity) represents the false positive rate, indicating the probability that the model incorrectly classifies surviving patients as deceased. The y-axis (Sensitivity) represents the true positive rate, indicating the probability that the model correctly classifies deceased patients as deceased. A larger AUC value indicates a stronger discriminative ability of the model.

Supplementary Figure 5 Immune infiltration analysis results via the xCell algorithm. **(A)** Infiltration proportions of 44 immune cell types in samples from the high-risk and low-risk cohorts. **(B)** Differences in the proportions of 44 immune cell types between the high-risk and low-risk cohorts. ns: p > 0.05; *: p< 0.05; **: p< 0.01; ***: p< 0.001; ****: p< 0.0001. **(C)** Correlations between differentially infiltrated immune cells and prognostic genes. The color indicates the correlation coefficient, with yellow representing positive correlation and blue representing negative correlation. Asterisks denote statistical significance: *: p< 0.05; **: p< 0.01; ***: p< 0.001.

Supplementary Figure 6 **(A, B)** nFeature_RNA, nCount_RNA, and percent.mt before and after quality control.

Supplementary Figure 7 **(A)** Acquisition of HVGs. **(B-D)** Selection of usable PCs. **(E)** Identification of cell types.

Supplementary Figure 8 Dimensionality reduction and clustering of the GSE132465 dataset. **(A)** Violin plots of the GSE132465 dataset after quality control (QC), including the number of genes detected per cell (Number of Genes), the Unique Molecular Identifier (UMI) count per cell, and the percentage of mitochondrial gene expression per cell (Percentage of MT). The x-axis represents samples. **(B)** Scatter plot of the top 2,000 highly variable genes (HVGs). The x-axis denotes the average expression level of genes (Average Expression), and the y-axis denotes the standardized variance of genes (Standardized Variance). **(C)** Principal Component Analysis (PCA) scatter plot of single-cell data before batch effect correction, where each scatter represents a single cell. **(D)** Jackstraw plot for statistical significance testing of Principal Components (PCs). The x-axis shows the theoretical P-value distribution, and the y-axis shows the actual P-value of each principal component. Each data point in the plot represents one principal component. **(E)** Elbow plot of PCA. The x-axis represents the serial number of PC sorted by variance explanation ability, and the y-axis represents the standard deviation corresponding to each principal component (Standard Deviation). (**F**) Uniform Manifold Approximation and Projection (UMAP) plot of cell clustering.
