# Supplementary figures and images for: The potential of mitochondrial permeability transition-driven necrosis-related genes in prognostic evaluation of colorectal cancer patients
Source: Front Oncol. 2026 Mar 9;16:1679360. doi: 10.3389/fonc.2026.1679360 (PMC13006312; doi:10.3389/fonc.2026.1679360)

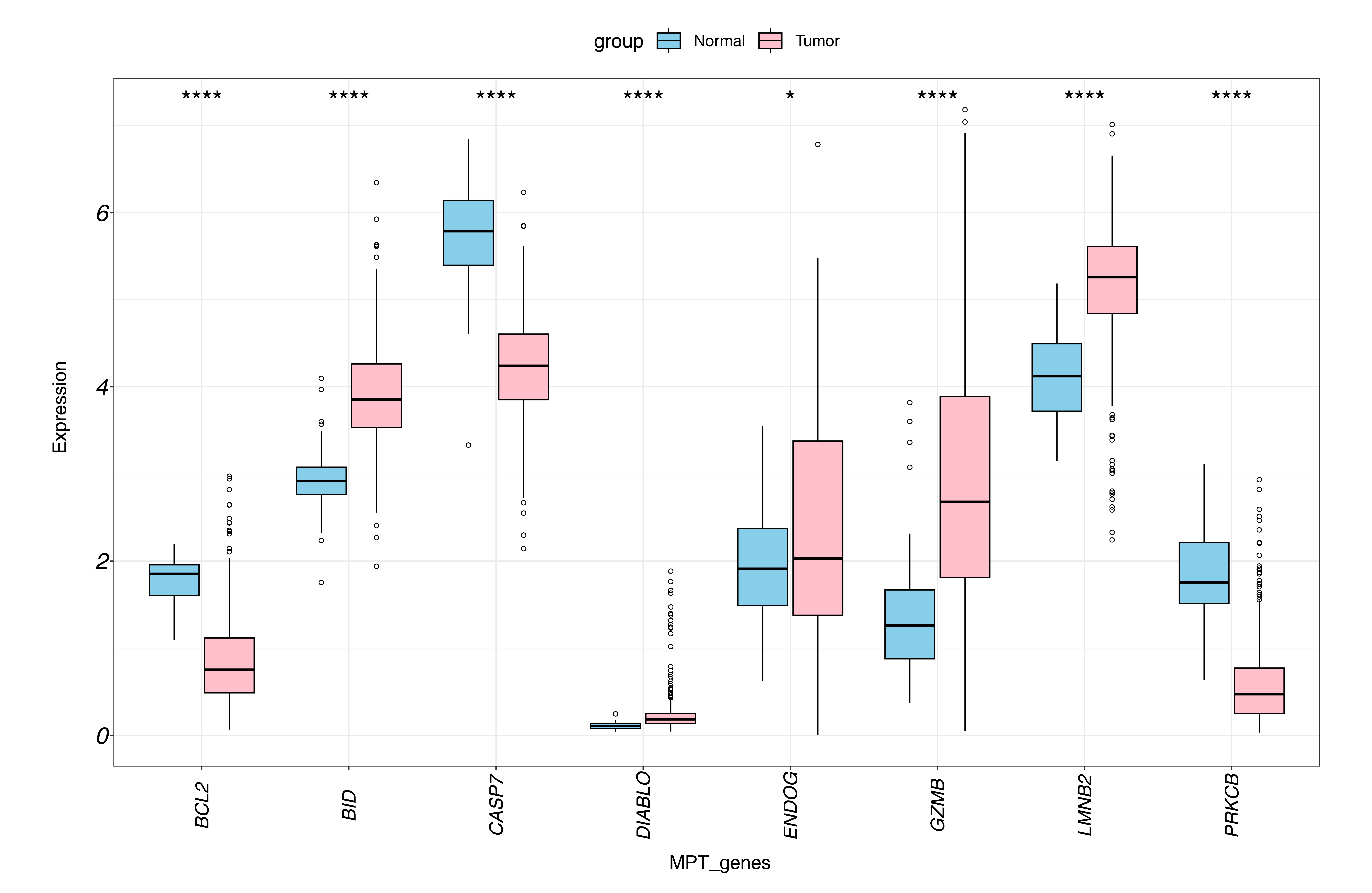

Supplement: Supplementary Figure 1 — Expression profiles of 8 de-mptdnrgs. [file Image1.tif]

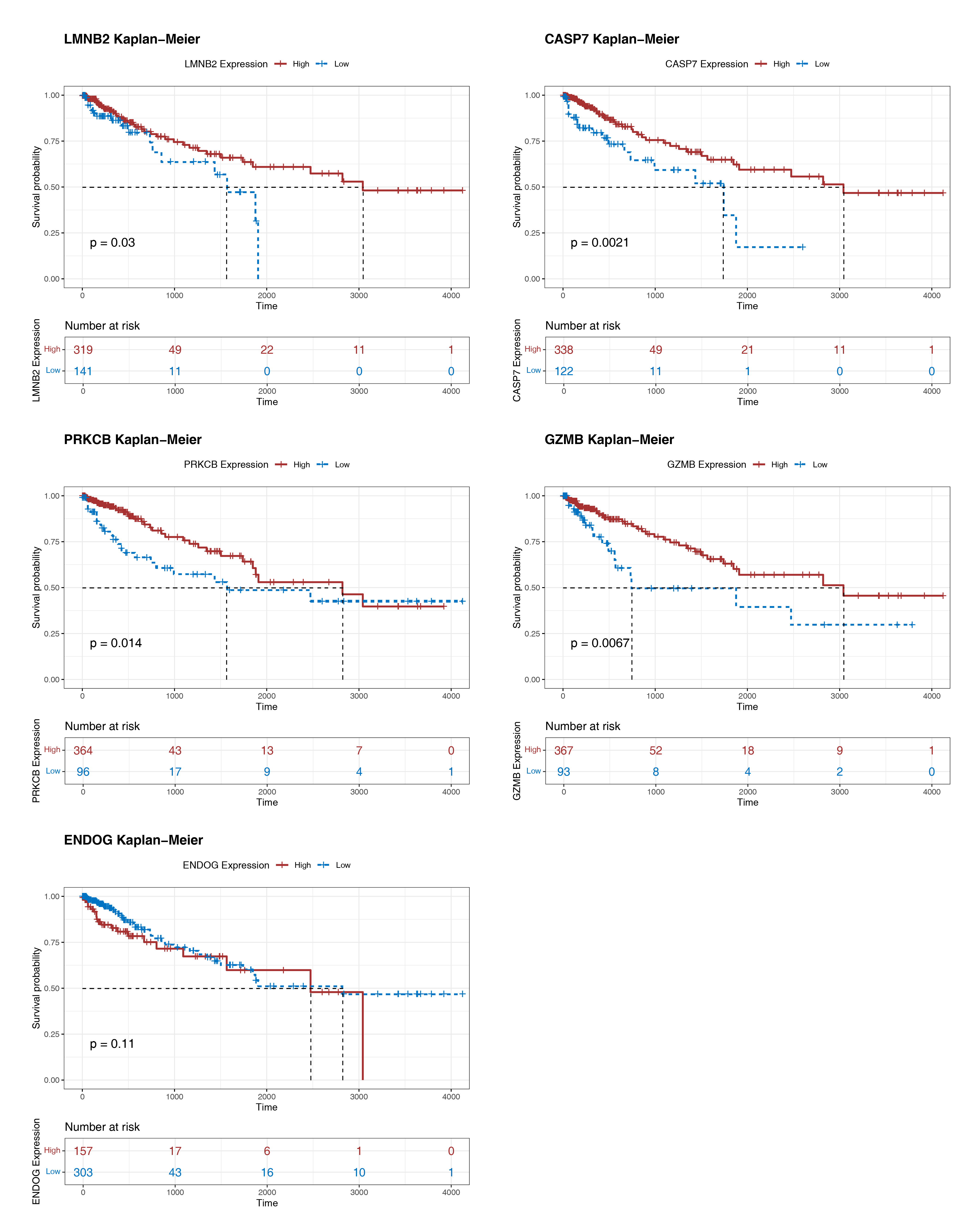

Supplement: Supplementary Figure 2 — Survival curves of the five prognostic genes. [file Image2.tif]

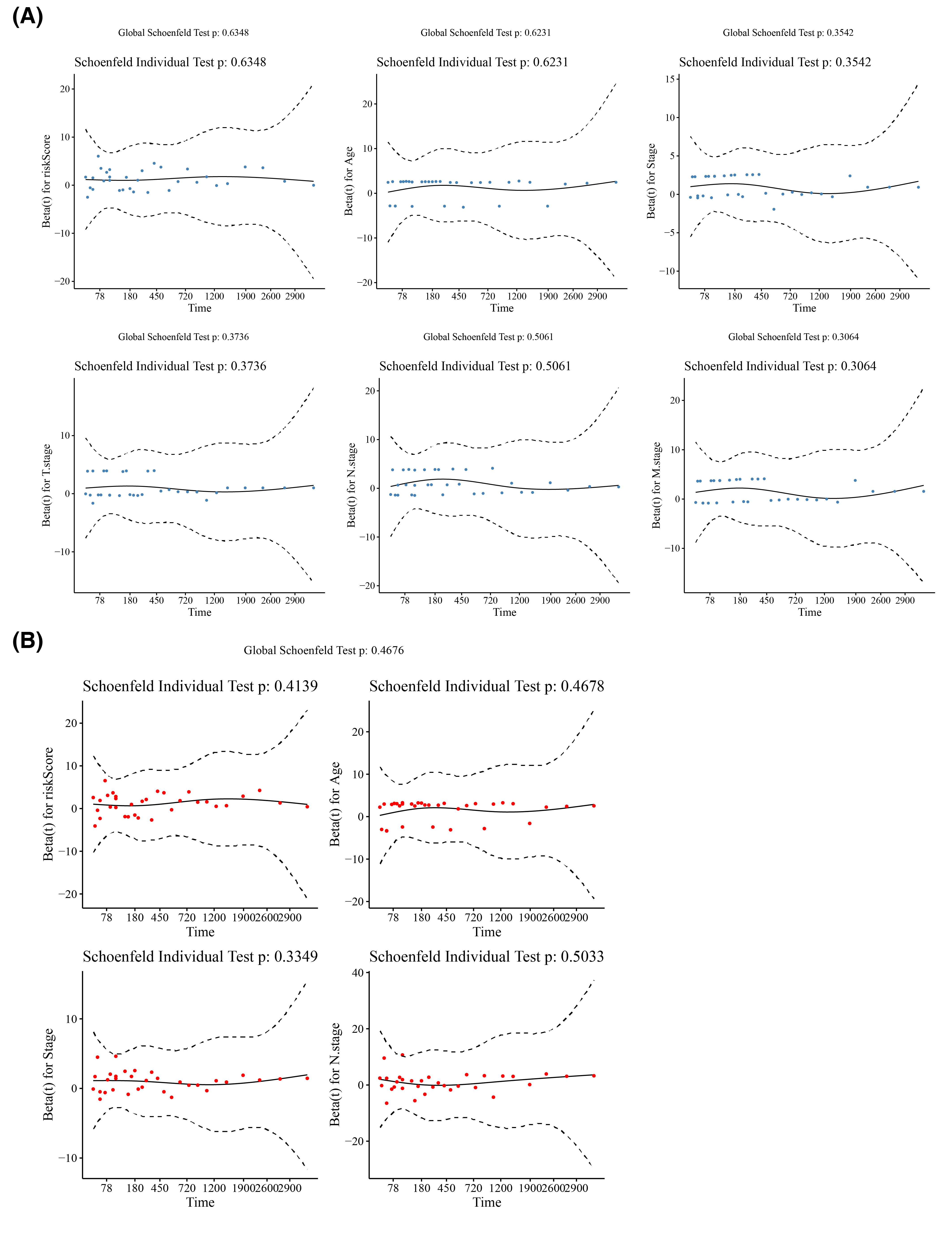

Supplement: Supplementary Figure 3 — Proportional Hazards (PH) Assumption Test Results for Independent Prognostic Analysis. (A) PH assumption test results of univariate Cox regression analysis for the risk score and clinical factors. The x-axis (Time) represents the survival time of patients. The y-axis (Beta (t) for riskScore) denotes the time-dependent changes in the regression coefficient corresponding to the risk score, reflecting the temporal dependency of the risk score’s impact intensity on survival risk. The regression coefficient curve in the figure shows no obvious upward or downward trend over the entire follow-up period and fluctuates around the value of 0, which intuitively confirms that the risk effect of the risk score does not change over time, further supporting the statistical conclusion that the proportional hazards assumption is satisfied. The proportional hazards assumption is considered to be met when the P-value is greater than 0.05. (B) PH assumption test of multivariate Cox regression analysis for the factors identified by univariate Cox regression analysis. [file Image3.tif]

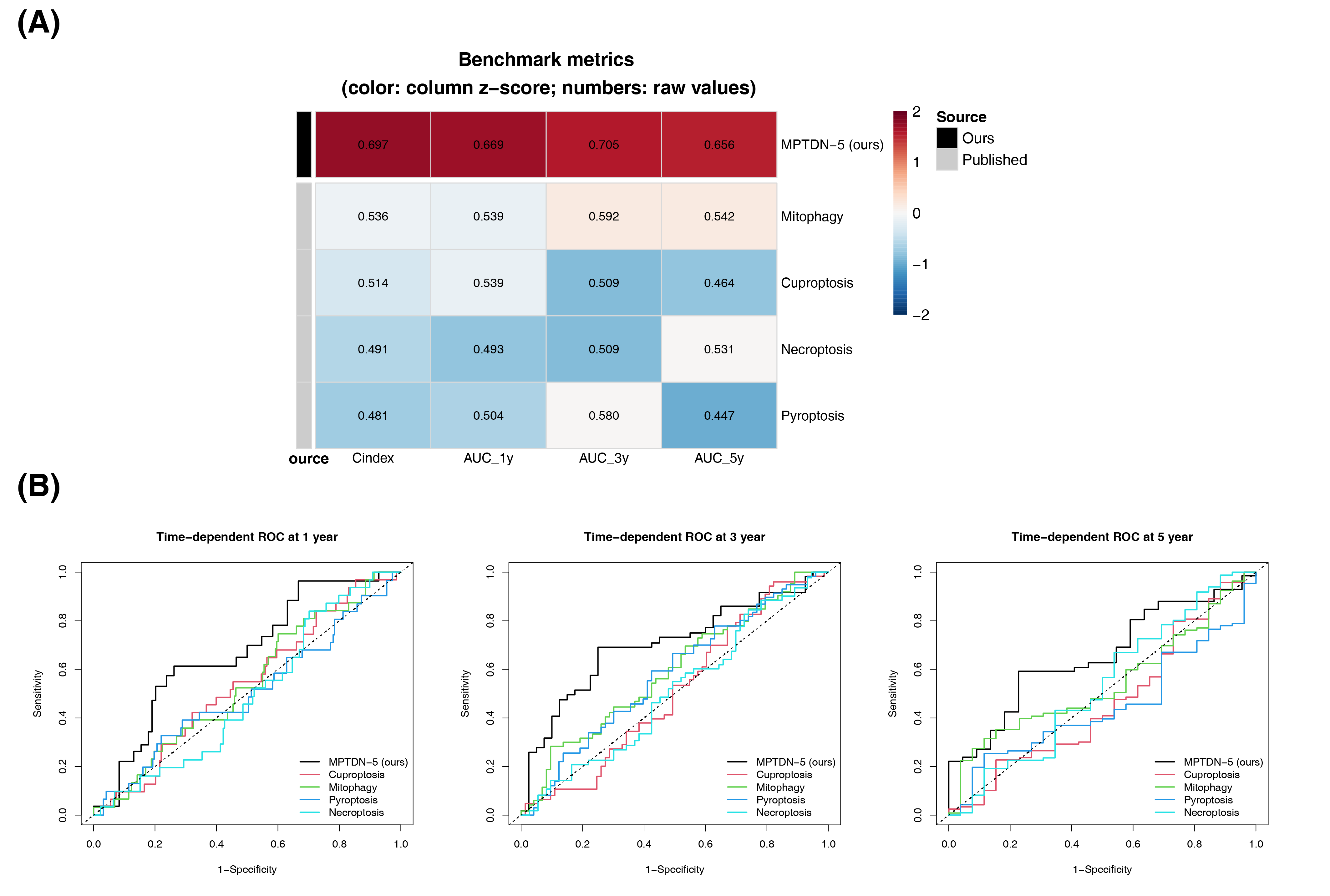

Supplement: Supplementary Figure 4 — Comparison of the prognostic model with published prognostic models. (A) Comparative analysis results of prognostic performance between the MPTDN-5 model (5 prognostic genes) constructed in this study and the published models. Rows include the prognostic gene model established in this study and four cell death-related subtypes, namely mitophagy, cuproptosis, necroptosis, and pyroptosis. Columns consist of five prognostic performance evaluation metrics, namely Concordance Index (Cindex), 1-year area under the curve (AUC_1y), 3-year area under the curve (AUC_3y), and 5-year area under the curve (AUC_5y). The numbers in the table are raw performance values, and the color of each column corresponds to the z-score normalized values. Published refers to previously reported prognostic models of the same category. (B) Time-dependent receiver operating characteristic (ROC) curves showing the performance of the MPTDN-5 model in predicting 1-year, 3-year, and 5-year survival outcomes of colorectal cancer (CRC) patients across four cell death-related subtypes (cuproptosis, mitophagy, pyroptosis, necroptosis). The x-axis (1−Specificity) represents the false positive rate, indicating the probability that the model incorrectly classifies surviving patients as deceased. The y-axis (Sensitivity) represents the true positive rate, indicating the probability that the model correctly classifies deceased patients as deceased. A larger AUC value indicates a stronger discriminative ability of the model. [file Image4.tif]

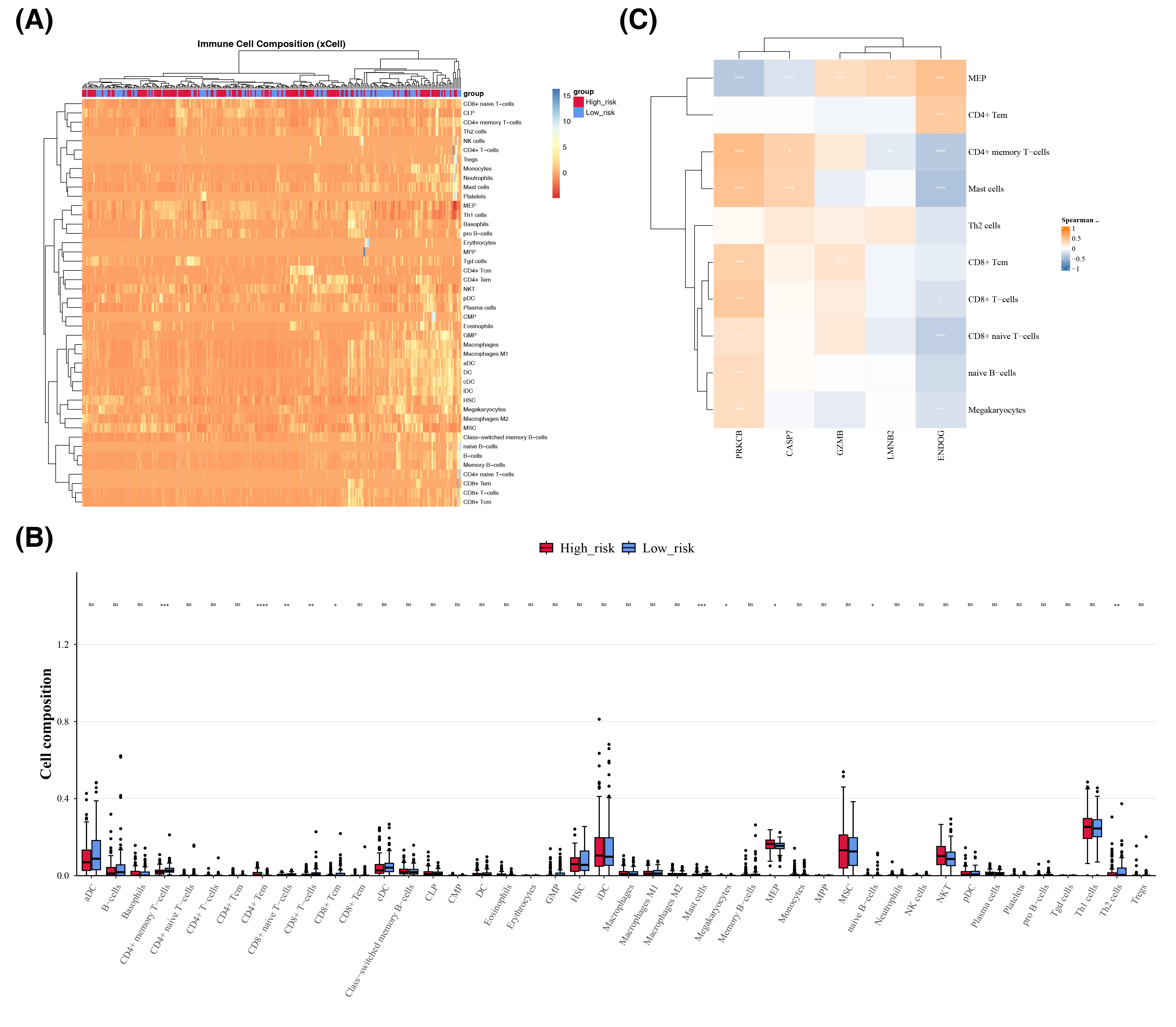

Supplement: Supplementary Figure 5 — Immune infiltration analysis results via the xCell algorithm. (A) Infiltration proportions of 44 immune cell types in samples from the high-risk and low-risk cohorts. (B) Differences in the proportions of 44 immune cell types between the high-risk and low-risk cohorts. ns: p > 0.05; *: p< 0.05; **: p< 0.01; ***: p< 0.001; ****: p< 0.0001. (C) Correlations between differentially infiltrated immune cells and prognostic genes. The color indicates the correlation coefficient, with yellow representing positive correlation and blue representing negative correlation. Asterisks denote statistical significance: *: p< 0.05; **: p< 0.01; ***: p< 0.001. [file Image5.tif]

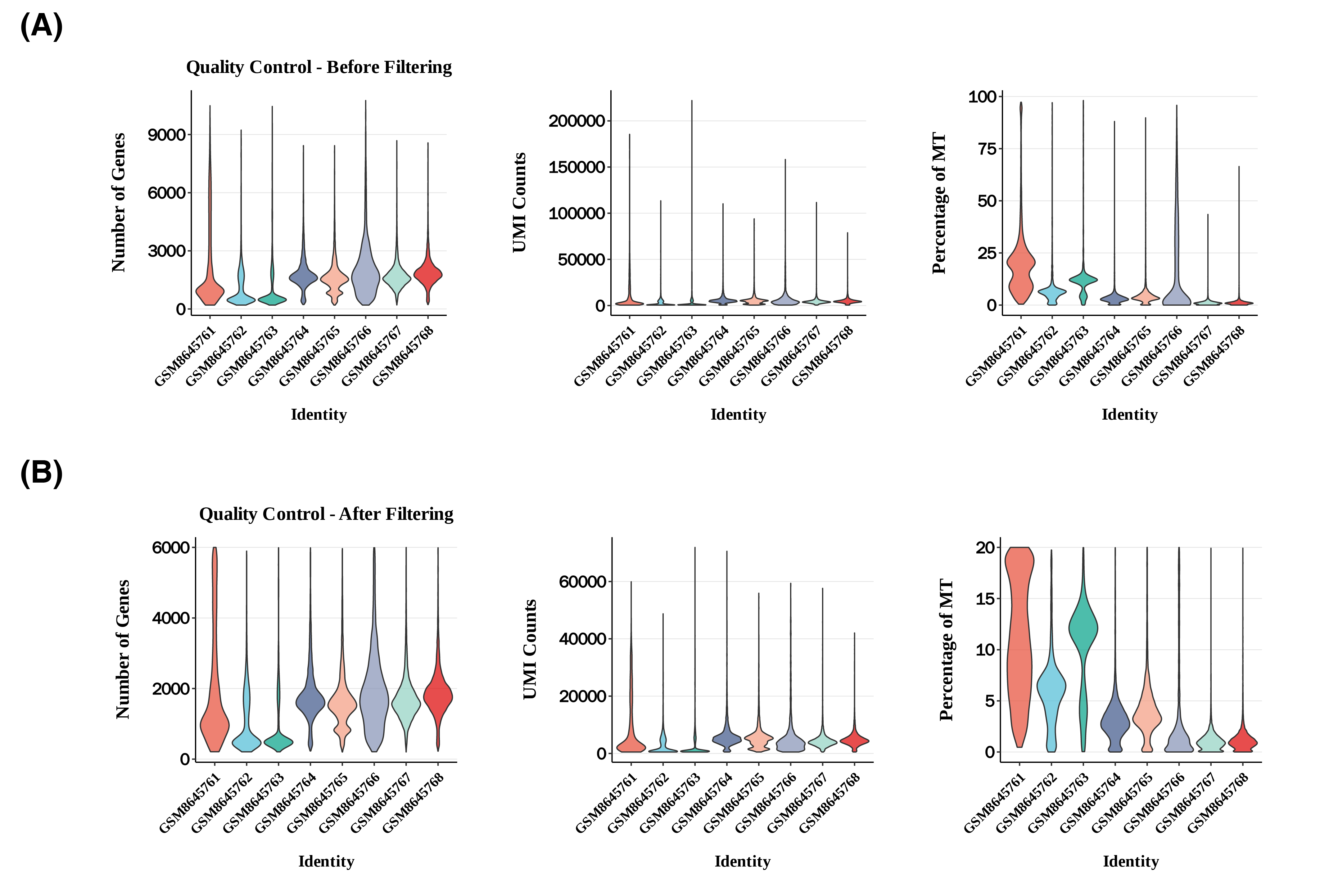

Supplement: Supplementary Figure 6 — (A, B) nFeature_RNA, nCount_RNA, and percent.mt before and after quality control. [file Image6.tif]

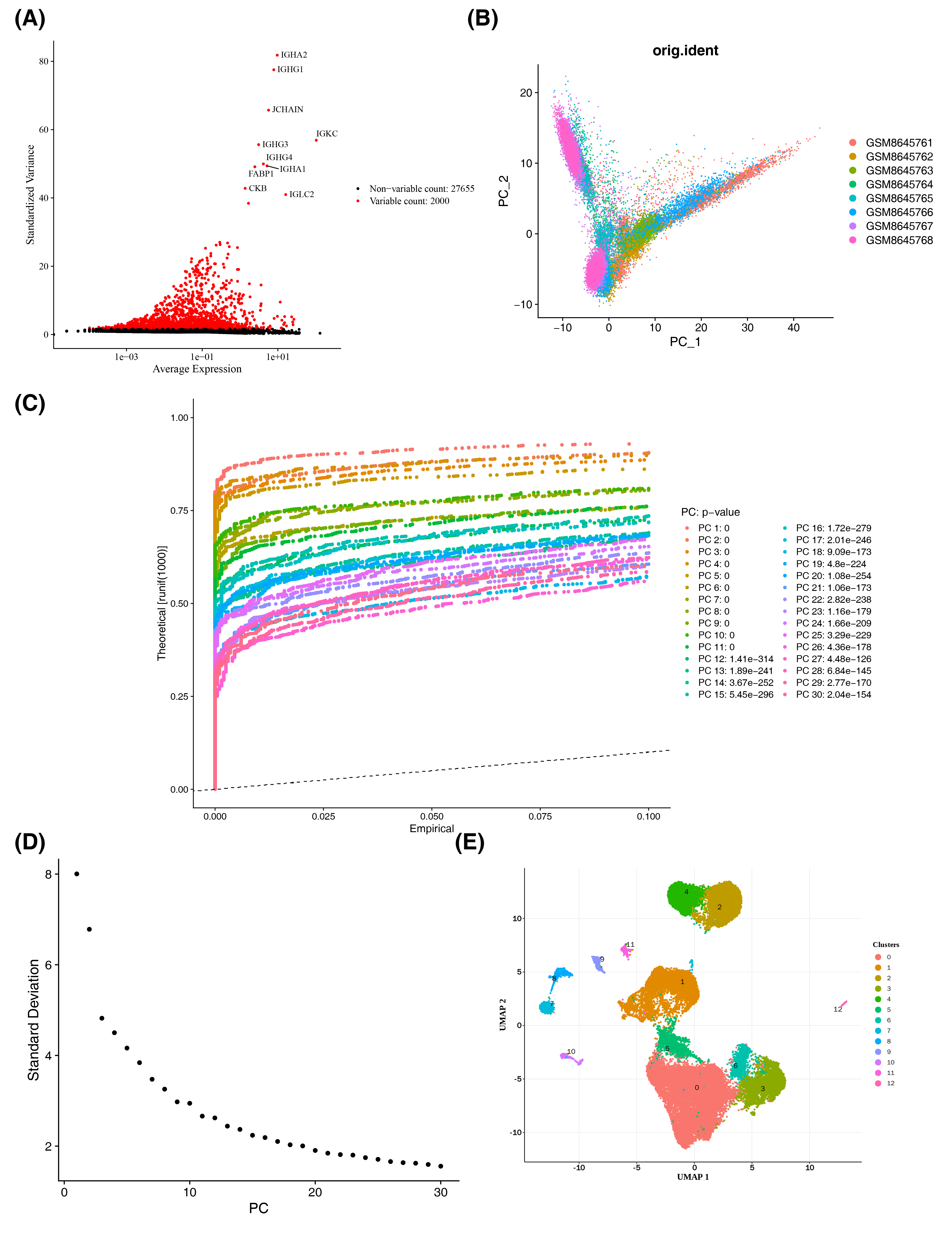

Supplement: Supplementary Figure 7 — (A) Acquisition of HVGs. (B-D) Selection of usable PCs. (E) Identification of cell types. [file Image7.tif]

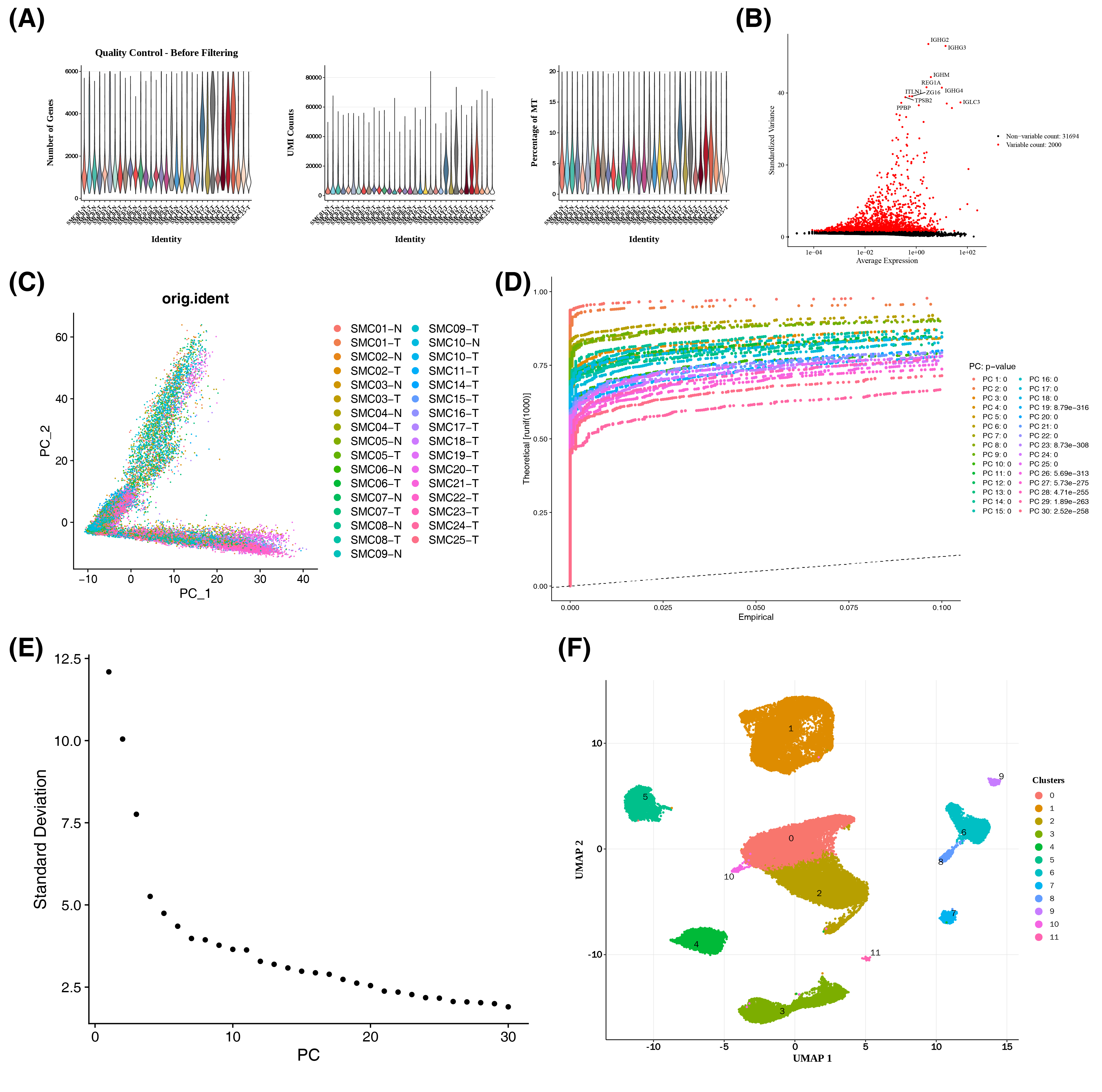

Supplement: Supplementary Figure 8 — Dimensionality reduction and clustering of the GSE132465 dataset. (A) Violin plots of the GSE132465 dataset after quality control (QC), including the number of genes detected per cell (Number of Genes), the Unique Molecular Identifier (UMI) count per cell, and the percentage of mitochondrial gene expression per cell (Percentage of MT). The x-axis represents samples. (B) Scatter plot of the top 2,000 highly variable genes (HVGs). The x-axis denotes the average expression level of genes (Average Expression), and the y-axis denotes the standardized variance of genes (Standardized Variance). (C) Principal Component Analysis (PCA) scatter plot of single-cell data before batch effect correction, where each scatter represents a single cell. (D) Jackstraw plot for statistical significance testing of Principal Components (PCs). The x-axis shows the theoretical P-value distribution, and the y-axis shows the actual P-value of each principal component. Each data point in the plot represents one principal component. (E) Elbow plot of PCA. The x-axis represents the serial number of PC sorted by variance explanation ability, and the y-axis represents the standard deviation corresponding to each principal component (Standard Deviation). (F) Uniform Manifold Approximation and Projection (UMAP) plot of cell clustering. [file Image8.tif]
